# Polymer Prolate Spheroids, Ellipsoids, and Their Assemblies at Interfaces—Current Status and Perspectives

**DOI:** 10.3390/ma19020291

**Published:** 2026-01-10

**Authors:** Damian Mickiewicz, Mariusz Gadzinowski, Stanislaw Slomkowski, Teresa Basinska

**Affiliations:** Division of Functional Polymers and Polymer Materials, Centre of Molecular and Macromolecular Studies, Polish Academy of Sciences, H. Sienkiewicza 112, 90-363 Lodz, Poland; damian.mickiewicz@cbmm.lodz.pl (D.M.); mariusz.gadzinowski@cbmm.lodz.pl (M.G.); stanislaw.slomkowski@cbmm.lodz.pl (S.S.)

**Keywords:** spheroidal particle, polymer spheroid, self-assembling, microspheroids, particle adsorption, nematic liquid crystal, colloidal crystal

## Abstract

Most nanoparticles and microparticles used as carriers of bioactive compounds are spherical in shape. Such particles are the easiest to obtain, as many processes spontaneously minimize the surface energy of the objects produced. However, in recent years, scientists have turned their attention to non-spherical particles in the hope of obtaining particles that interact with their environment in a tailored manner. The production of such particles should be easy and reproducible. The best candidates are spheroids produced by various methods. The most often used is the linear transformation of spheres during processes that preserve constant particle volume. The typical process consists of stretching a polymer matrix filled with spherical particles. The article delivers a critical overview of methods, discussing their advantages and disadvantages. A list of presented methods also includes the preparation of spheroids by polymer solution emulsification-solvent evaporation, controlled dispersion polymerization, electrohydrodynamic jetting, adsorption of amphiphilic copolymers on solid particles, and copolymer self-organization processes, as well as microfluidic methods, deformation of spherical particles into spheroids by irradiation, and phase microseparation. A special section is devoted to the self-organization of the particles at the phase boundaries. Eventually, the preparation and selected properties of two-dimensional and three-dimensional assemblies of spheroidal particles, particularly the preparation of a quasi-nematic colloidal crystal, are discussed.

## 1. Introduction

A brief comment on terminology: According to IUPAC recommendations, nanospheres are particles with diameters ranging from 1 to 100 nm, whereas microspheres are particles with diameters ranging from 100 nm to 100 μm [1].

Research on the synthesis, properties, and forced and spontaneous organization of spherical, colloidal polymer particles has been performed for several decades. The studies have led to the development of industrial methods for the manufacturing of polymers (e.g., polystyrene and polyacrylates) on a large scale. However, there is also a large number of classes of special spherical particles designated for application in medicine, cosmetics, agriculture, sensors, carriers of immobilized catalysts (including enzymes), environmental protection, and many others. Research in this field remains very intensive, as evidenced by the large number of recent publications. According to the Web of Science database, over the past five years, this number has reached over forty-five thousand; the vast majority of investigated particles were spherical. Mention should be made not only of the growing interest in suspensions of nanoparticles and microparticles but also of their ordered assemblies, in particular the so-called colloidal photonic crystals. The latter are built of periodically arranged spherical particles of identical diameters packed into regular networks. Their structure is similar to that of the crystal network. The main difference lies in the size of the objects placed at the nodes of the crystalline networks. The photonic crystals transmit and reflect light in predetermined directions, and the maximum intensity of the reflected wavelength depends on the diameter of the particle [2,3]. The polymer particle-based photonic crystals have already found applications in light-conducting materials, sensors responding to environmental stimuli (pH, biologically active compounds, etc.) [4,5,6]. The efficient formation of colloidal crystals requires an understanding of the mechanisms of controlled organization and spontaneous self-organization of particles. This knowledge is practical for designing modern materials and devices used in life sciences [7]. However, it is worth noting that photonic crystals, even if they have been formed as a result of perfect particle organization, have some disadvantages, such as an incomplete band gap resulting from symmetry-induced degeneration of the crystal lattice [8,9,10,11,12,13,14].

**Figure 1:**
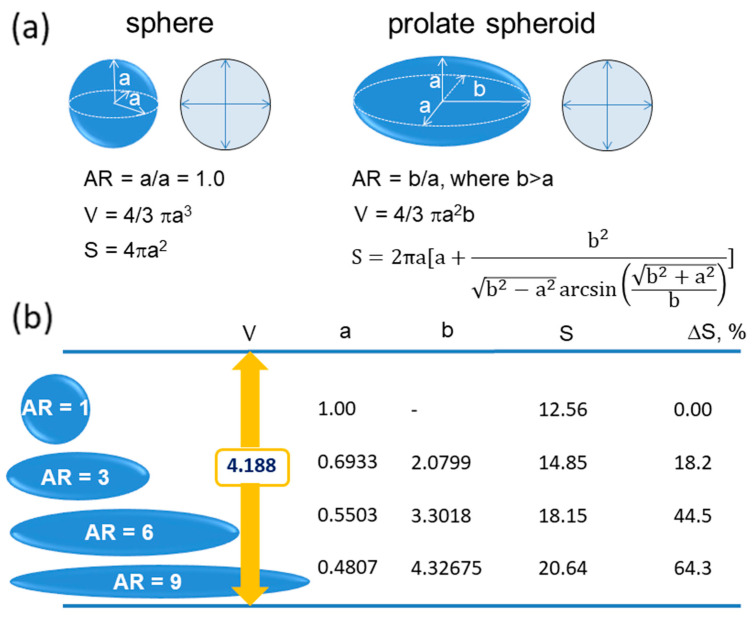
Spatial properties of spherical and prolate spheroidal particles: (**a**) cross-sections and equations for calculating aspect ratio (AR), surface (S), and volume (V), (**b**) comparison of short and long axes and surface of the spherical and prolate spheroidal particles with the same volumes (V = 4.188, arbitrary units).

The properties of polymer particles depend on their size and the chemical composition of the core and interfacial layers. Particles with the required properties are usually obtained by controlled synthesis or post-synthesis chemical modification. However, there is also another option for preparing particles with new properties. This method involves replacing spherical particles with anisotropic ones [15]. The simplest are prolate spheroidal particles. Prolate spheroidal particles are defined as particles produced by uniaxial elongation of spherical particles, provided that during deformation their volumes are maintained constant. The geometry of the prolate spheroidal particle is defined by the length of the two mutually perpendicular short axes of the length *a* (each perpendicular to the direction of the particle elongation) and the long axis *b* directed parallel to elongation. The *b*/*a* ratio is defined as the aspect ratio and abbreviated as AR (see Figure 1).

It is worth noting that literature data contain numerous papers on bio-organic objects like proteins, lipids, oligosaccharides and viruses with spheroidal shape. However, the papers do not contain precise information on the shape of the objects that they cannot be considered as geometrical “spheroids”.

The prolate spheroidal and spherical particles differ in two important features. Namely, the first is that for particles of the same volume, the surface area of spheroidal particles is always larger than the surface area of spherical particles. The second is that the curvature of the surface of spherical particles is equal at every location, while the curvature of the surface of spheroidal particles increases at their ends. These features and their consequences are discussed in more detail below. Comparison of the spatial characteristics of spheres and spheroids is shown in Figure 1.

As it has been mentioned, according to basic geometry, for particles of the same volume, the surface area of spheroidal particles is larger than that of spherical particles. Thus, it is reasonable to assume that the spheroidal particles will interact more efficiently with their surroundings.

Moreover, chemical composition and electric charge density in the interfacial layer of spherical particles are distributed in an isotropic manner. For the spheroidal particles, the charge density and electric potential at the sharp particle ends should be different from those at the other particle’s surface parts. A worth mentioning consequence of the various shapes of spherical and spheroidal particles is the difference in the number of their degrees of freedom in space. A spherical particle has only three defining its position in space, while a spheroidal particle has two more, due to the angular orientation of the particle’s long axis. Therefore, spheroidal particles have more possibilities for creating new types of particle assemblies.

Although the potential for using spheroidal particles has been recognized, knowledge of their production and properties is rather modest. Typically, polymer spheroids are obtained from previously synthesized spherical particles with carefully planned morphology, diameters, and surface properties. The spherical particles are then uniaxially elongated [15]. For this purpose, the spherical particles are embedded in a water-soluble polymer film, which is subjected to elongation to the required film length at a controlled temperature and time. The prolate particles formed in the film are isolated by dissolving it. Microfluidic methods, on the other hand, make it possible to obtain spheroidal particles under properly selected flow conditions in a direct way.

This paper presents a review of the results of previous and recent studies on the methods of manufacturing polymer spheroidal particles with a broad range of aspect ratios and morphologies. There are also studies of the manufacturing of ordered systems from elongated spheroidal microparticles, and studies into the dependence of microparticle self-organization in mono- and multilayer systems on various solid substrates. In particular, the spontaneous self-organization of the prolate spheroidal particles occurs without the action of any external forces, such as electric or magnetic fields.

## 2. Preparation of Polymer Nano- and Microspheroids

Commonly used emulsion and dispersion polymerization methods produce suspensions of polymer nano- or microspheres. The microsphere integrity is ensured by cohesion forces between the polymer macromolecules, and their spherical shape results from minimizing the surface free energy per particle. For particles of the same polymer, the surface energy is lowest for the microspheres.

**Figure 2:**
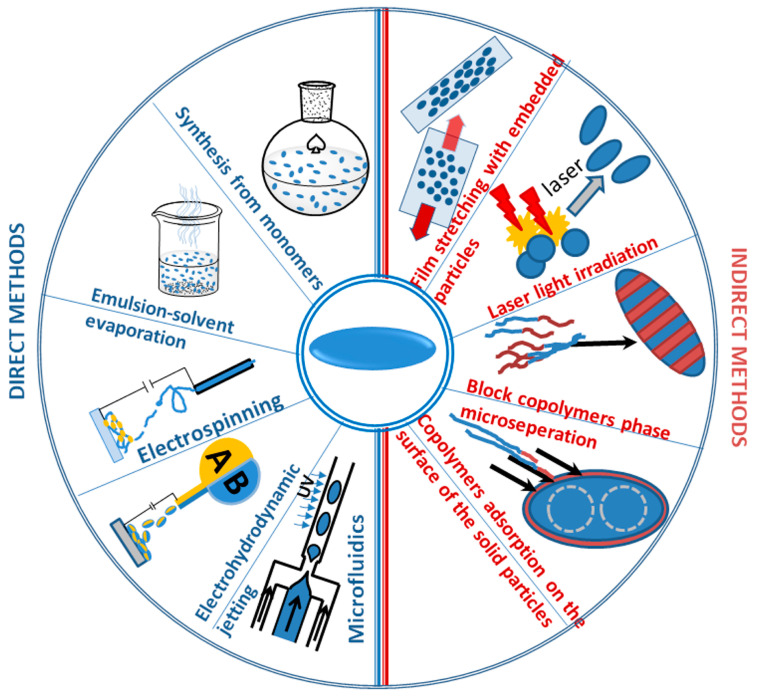
Overview of the methods used to produce the polymeric spheroidal particles. Reprinted with permission from the ref. [17].

From the 1960s, the most common methods of preparing prolate spheroidal particles were mechanical uniaxial deformation of polymer microspheres carried out above their glass transition [16] and various other combinations of chemical and physical processes (see Figure 2), which include:

(1) methods involving controlled deformation of spherical particles:

- stretching a polymer matrix with particles [15]; the type of matrix and the method of immobilization of particles in matrices depend on the polymer properties of the particles;

(2) methods employing direct formation of the spheroidal particles (formed “in situ”):

- direct synthesis of elongated particles from the monomers;
- emulsion-solvent evaporation of the polymers/copolymers;
- electrospinning; microfluidic methods.

### 2.1. Obtaining Spheroids by Stretching a Polymer Matrix Filled with Spherical Particles

In most cases, the prolate spheroidal particles with a controlled aspect ratio are obtained by deforming the microspheres. The oldest known and still used method is the uniaxial stretching of an elastomer film with embedded polymer microspheres [18,19]. Commonly used elastomers are poly(vinyl alcohol) (PVA) [18,19] and poly(dimethylsiloxane) (PDMS) based mixtures with methylhydro/dimethyl siloxane copolymer (PMH/PDMS) [20]. PVA is dissolved in water (usually at a concentration of about 10%) and then mixed with an aqueous suspension of the particles. This mixture is placed in molds. After drying, a film of PVA filled with spherical particles is formed. The film is then stretched to the required length at a controlled rate, and at a temperature above the glass transition (T_g_) of PVA and of the polymer/s used for the preparation of the particle. The film is then dissolved in water, and the elongated particles are recovered by centrifugation and purified by repeated resuspension in pure water and isolated by centrifugation [18,19,21]. Cross-linked polysiloxane strips containing poly(methyl methacrylate) microspheres were prepared by mixing a suspension of microspheres in hexane with a viscous hexane solution of PDMS, PMH/PDMS, and tin 2-ethylhexanoate catalyst, and cross-linking this mixture in a mold. After stretching the strips to the required length, the strips underwent degradation by sodium or potassium methoxide solutions to isolate the elongated particles from the film [20].

**Figure 3:**
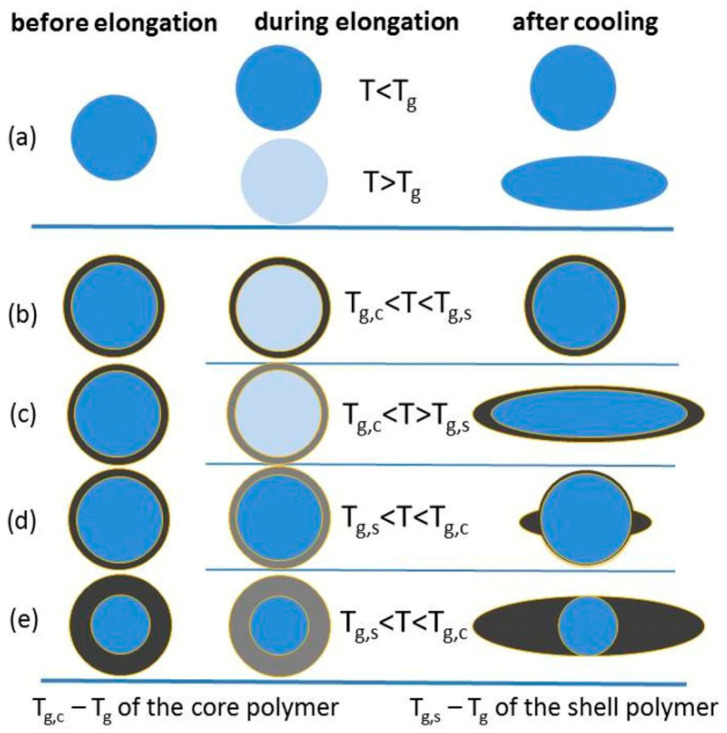
The shape and morphology of the homogenous and core–shell particles, which underwent uniaxial stretching of the films with embedded microspheres in various T of elongation: (**a**) homogenous particles, (**b**–**e**) core–shell particles. The darker and lighter colors of the particle core/shell indicate the polymer below and above T_g_, respectively.

A schematic illustration of described in the literature various shapes of homogenous and core–shell particles formed by stretching elastomer strips with embedded microspheres is shown in Figure 3 [22,23,24].

The uniaxial elongation of a homogenous polymer particle can lead to shape deformation and the formation of a spheroid, if the temperature of the film stretching is higher than the glass transition temperature of the particle polymer (as presented in Figure 3a).

In the case of microspheres with core–shell morphology, the strip elongation process leads to the formation of particles with more complex morphology, as illustrated in Figure 3b–e.

For particles with the core–shell morphology, the following possibilities should be considered. When the glass temperature of the shell (T_g,s_) is higher than the core glass temperature (T_g,c_), raising the temperature to T_g,c_ < T < T_g,s_ would result in particles with soft semifluid cores and glassy shells (as shown in Figure 3b). Such particles cannot be elongated under mechanical forces and return to their original state upon cooling. When T_g,c_ ≈ T_g,s,_ and the thermomechanical properties of the core and shell polymers are similar, the deformation of spherical homogeneous particles and core–shell particles, embedded in a stretched elastomer strip, proceeds similarly, yielding spheroids (as is visible in Figure 3c). A completely different particle shape appears after the deformation of the spherical core–shell particles with T_g,s_ < T < T_g,c_ performed at a temperature between these glass transition temperatures (T_g,s_ < T < T_g,c_). At the mentioned temperature, the core remains in a glassy state. It therefore cannot undergo any deformation, while the polymer in the shell can be deformed above the glass transition temperature, making the entire deformation process complex (see Figure 3d) [22]. In the case of elongation of the microspheres with a thin shell, the elongated particles containing spherical cores are obtained, as illustrated in Figure 3e.

Attention should also be paid to the situation when the matrix elongation is too fast, which can lead to cavitation and loss of control over microsphere elongation.

Obtaining spheroidal particles by stretching PVA films with embedded spherical particles has been known since the 1960s, when Felder obtained spheroids with aspect ratios of 5 and 10 from poly(vinyl acetate) (PVAc) [16]. In the 1980s, Keville et al. used a matrix of cross-linked PDMS to obtain spheroids from poly(4-vinyltoluene) and poly(methyl methacrylate) with aspect ratios in the range of 1.7–7.7 [18]. It is worth mentioning that the authors presented a complete description of obtaining spheroidal particles using cross-linked PDMS as a matrix in 1993. Ho et al. developed a method for achieving polystyrene spheroids with a preset aspect ratio in the range of 1.93–5.65 and a uniform degree of elongation [21]. PVA film was used as a matrix in this process. The aforementioned method consisted of placing strips of film containing microspheres between clamps immersed in oil. During stretching, the oil was maintained at the required temperature. The elongation of the strips controlled the elongation of the particles. After 2000, intensive research on anisotropic particles continued. In 2007, Champion et al. published the results of a study on modifying an existing method for producing the polymer particles with shape anisotropy [22]. Using polystyrene microspheres with diameters ranging from 0.06 to 10 µm, particles of various shapes were obtained. In addition, the authors found that in the case of uniaxial stretching of PVA films, the particle shape is affected by the film thickness. When, as a result of uniaxial strip elongation, the film thickness decreased to about 20 times the diameter of the deposited microspheres, the particles flattened, becoming ellipsoidal (i.e., particles with an elliptical cross-section). On the other hand, when the ratio of film thickness to microsphere diameter was equal to or exceeded 40, spheroidal particles (i.e., particles with a circular cross-section) were obtained. Therefore, since a decrease in film thickness accompanies the film elongation, the preparation of large spheroidal particles with a high aspect ratio requires films with a sufficiently large initial film thickness.

It should also be noted that the methods mentioned above can also be used to prepare hollow spheroidal particles. For example, Xu et al. used a commercially available dispersion of hollow polystyrene microspheres with an average diameter of *D*_n_ = 430 nm for uniaxial elongation under controlled conditions [23]. However, particle disintegration was observed after exceeding a critical length of 300% of the initial microsphere diameter.

Recently, the uniaxial film stretching technique has been extended to produce spheroids with a wide and controlled aspect ratio in a one-step process by gradient stretching of a strip of PVA film with embedded polystyrene particles [24]. The results showed that by using an intended and calibrated simultaneous gradient of film elongation and subsequent cooling, spheroids with the designed aspect ratio spectrum can be produced from spherical particles of a single size. The method has been successfully tested using polystyrene particles with a wide range of particle diameters, from 500 nm to 10 µm.

Moreover, spheroidal particles (as one fraction of a mixture of multi-shaped particles) can be obtained by multi-axial stretching of a PVA film disk [25]. The authors used clamps spaced along the perimeter of the PVA film disk. The disadvantage of this method is low efficiency—particles of different shapes are formed in various parts of the film, depending on the distance from the film center. Elongated spheroids can be isolated from a relatively small area of the film.

Attempts have also been made to obtain spheroidal-type particles by using multiaxial stretching of microsphere-embedded film through blowing [26]. However, it has not been easy to apply multiaxial stretching/blowing methods in a properly controlled manner.

### 2.2. Requirements for the Preparation of “Perfect” Spheroids by the Film Stretching Method

Authors of papers devoted to obtaining and using so-called spheroidal particles very rarely checked whether the process and product met the criteria to use this term. Namely, (i) the particle should be uniformly stretched in the polymer matrix, i.e., dividing the particle into regular slices—each slice of the particle (perpendicular to the direction of elongation) should be stretched to the same length, (ii) the density of the polymer in the particle (core and shell, if any) does not change during stretching (i.e., no crystallization process during stretching).

**Figure 4:**
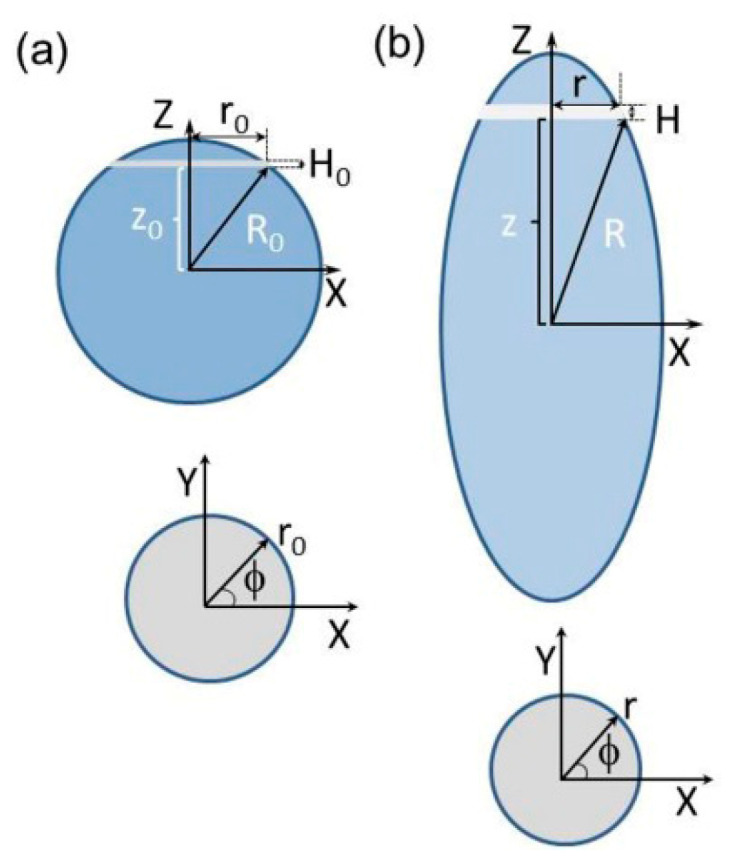
(**a**) Spherical particle and (**b**) prolate spheroid in cylindrical coordinates. The subscript “0” is used for the spherical particle.

**Figure 5:**
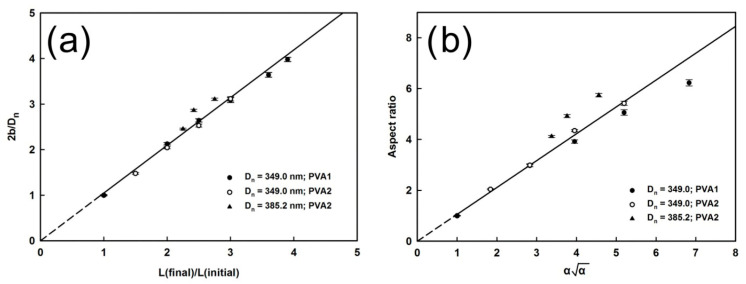
Relations between microspheroid and film stretching: (**a**) dependence of the microparticle and the strip of the film with embedded particles stretching; D_n_ denotes number average diameter of microparticle; ratio L(final)/L(initial) denotes length of the film strip after and before elongation, respectively; (**b**) dependence of the aspect ratio of PS/PGL microspheroids and the ratio of the final to the initial length of the elongated particle. Reprinted with permission from the ref. [27].

According to the literature, this issue was first addressed by Komar et al., who synthesized microspheres with a polystyrene core and polyglycidol-enriched shell and subjected them to uniaxial elongation in a PVA matrix [27]. To answer the question of whether the particles are “true spheroids,” they analyzed the geometry of the uniaxial deformation of the spherical particles obtained by stretching (see Figure 4) and established the relationship between the stretching of the film and the final dimensions of the resulting microspheres (the corresponding graphs are shown in Figure 5a,b).

In Figure 4, R_0_ denotes the radius of the microsphere, z_0_ the position of an arbitrarily chosen virtual thin “slice” of the microsphere, H_0_ the thickness, and r_0_ the diameter of the slice. The relevant parameters without subscripts are used for the spheroid.

Denoting the elongation parameter as α (H = αH_0_, z = αz_0_) and remembering that deformation of the particles (and any parts thereof) does not change their volume (V_0_ = V, where V_0_, and V denote volume of the slice of the microsphere and the spheroid, respectively), the following relations should hold (Equations (1)–(4)):

(1) $$V_{0}=\pi r_{0}^{2}H_{0}$$

(2) $$V=\pi r^{2}H$$

(3) $$R_{0}^{2}H_{0}=r^{2}H$$

(4) $$R_{0}^{2}H_{0}=r^{2}\alpha H_{0}$$

(5) $$R_{0}=\sqrt{\alpha }r$$

and therefore, Equation (6)

(6) $$R_{0}^{2}=r_{0}^{2}+z_{0}^{2}$$

can be converted to

(7) $$1=\frac{r^{2}}{{\left(\frac{R_{0}}{\sqrt{\alpha }}\right)}^{2}}+\frac{z^{2}}{{\left(R_{0}\alpha \right)}^{2}}$$

(8) $$a=\frac{R_{0}}{\sqrt{\alpha }},b=R_{0}\alpha ,aspect\;ratio\;AR=\frac{b}{a}=\alpha \sqrt{\alpha }$$

Equation (7) corresponds to the projection of the contour of the elongated microsphere on the r, z plane of cylindrical coordinates. The form of Equation (7) indicates that it is an ellipse with short and long axes a and b, respectively. The values r, z, and ϕ (0 < ϕ ≤ 2π) define the position of the points on the surface of spheroidal particles in cylindrical coordinates, R_0_ denotes radius of the microsphere and means elongation of the microsphere, i.e., the ratio of the long axis of the microspheroid and radius of the microsphere (b/R_0_, see Figure 1 and Figure 4).

Figure 5a shows the relation between the number average of elongation of the microsphere (2b_n_/D_0n_ = b_n_/R_0,n_) and elongation of the PVA strip (L/L_0_) containing the dispersed particles. It is worth noting that the experimental points can be well fitted by a straight line with a slope of 1.05 ± 0.01. This means that controlled elongation of the PVA strip allows controlled elongation of the microspheres.

To check whether the discussed prolate microparticles with a polystyrene core and polyglycidol-enriched shell structure were the “true microspheroids”, the authors prepared a plot of the averaged aspect ratio (AR) as a function of $\alpha \sqrt{\alpha }$ (see Figure 5b). According to Equation (8), this plot should be represented by a straight line with slope 1. Experimental data fitted with a straight line yielded a slope of 1.06 ± 0.04, indicating that formed particles were perfect spheroids.

Recently, studies of elongation of negatively and positively charged polystyrene particles in PVA film showed similar results for the microspheres with diameters in the range from 340 to 1400 nm [28].

### 2.3. Spheroids Obtained by Emulsification-Solvent Evaporation

Usually, an oil-in-water emulsification solvent evaporation method yields spherical polymer particles. The process consists of emulsifying a hydrophobic polymer or copolymer (e.g., polylactide or poly(lactide-co-glycolide)) dissolved in an organic volatile solvent (e.g., methylene dichloride) in water and subsequent organic solvent evaporation. The water phase usually contains surfactants used as emulsion stabilizers. An addition of appropriate hydrophobic compounds to the polymer solution enables the preparation of particles loaded with dyes, drugs, and other bioactive compounds.

However, it is worth noting that adjusting the emulsion components and changing the hydrophilicity of the copolymer make it possible to obtain spheroids [29]. To prepare the particles, the authors used poly(lactic-co-glycolic acid) (PLGA) with chains terminated by carboxyl groups and an alkaline (pH = 8.4) aqueous phase containing 1% PVA and 1.2% Tris.

Studies have shown that the high viscosity of the aqueous phase, its alkalinity, the hydrophilic side chains of the polymer/copolymer, and the carboxyl ionic end groups promote the formation of the spheroidal particles. This method is particularly suitable for producing spheroidal particles from biodegradable polymers loaded with biologically active compounds applied in drug delivery systems. Unfortunately, control of the proportion of spheroids is limited. However, the method is simple, and spheroids can be produced with high yields in a relatively short time. The size and shape of the spheroid can be controlled to some extent by the emulsification rate, viscosity, and pH of the aqueous phase, the viscosity of the organic solvent (concentration and molecular weight of the polymer/copolymer used), and the fraction of hydroxyl and carboxyl end groups of the polymer/copolymer chains [29]. Cao at al. enriched the surface of the PLGA spheroidal particles in carboxyl groups by using poly(ethylene-alt-maleic acid) as polymer film matrix instead of PVA in particles elongation process [30]. The carboxyl groups were suitable to efficiently bind ligands recognizing receptors on the surface of the tumor cells.

### 2.4. Spheroids Obtained by Controlled Dispersion Polymerization

According to the literature data, the group of Tian et al. was the first to obtain spheroidal particles directly by polymerization [31]. Using a controlled radical polymerization (RAFT), the spheroidal particles from poly(glycidyl methacrylate) (PGMA) with a number-average molar mass of less than 5960 g/mol and an *M*_w_/*M*_n_ equal to 1.27 were manufactured. These values were much lower than those obtained by conventional dispersion polymerization (*M*_n_ = 27,640 g/mol and *M*_w_/*M*_n_ = 2.87 in the control sample). The authors used azo-bis-isobutyro-dinitrile (AIBN) as the initiator, and S-1-dodecyl-S′-(α,α′-dimethyl-α″-acetic acid) trithiocarbonate (DDMAT) as the agent to control the polymerization. In addition, the system contained polyvinylpyrrolidone (PVP) as a dispersing agent. During polymerization carried out at 70 °C, spherical particles were initially formed. However, after 2 h, it was observed that some of the particles present in the system were spheroidal in shape. In addition, the ratio of spheroids to spheres, as well as the particles degree of particle elongation, increased as the polymerization process progressed. After polymerization was complete, exclusively spheroidal particles were present in the suspension. The reason is attributed to the increasing viscosity of the mixture during the particle synthesis and the associated increasing shear forces. Under the influence of these forces, changing the shape of the particles is easier for shorter polymer chains. Finally, microspheroids acquired an average length-to-width ratio of 3.18, but with a wide scatter in the degree of elongation. In control experiments, it was confirmed that DDMAT, an agent used in controlled RAFT polymerization, is crucial in the process of spheroid formation and the initiation of polymerization of styrene from the particles’ surface. In consequence, spheroidal particles with a core–shell morphology were produced [31].

An important advantage of the method presented above is the possibility of producing very quickly a large number of spheroidal particles with an aspect ratio in the range of 1.00–3.18, depending on the monomer conversion. However, it should be added that there is no information in the literature on the direct synthesis of spheroidal particles from monomers other than glycidyl methacrylate. Nothing is also known about the formation of particles with an average aspect ratio exceeding 3.18, as well as the studies of the surface morphology of the synthesized core–shell spheroidal particles.

### 2.5. Spheroids Obtained by Electrohydrodynamic Jetting

Techniques called electrohydrodynamic jetting involve pumping electrically charged streams of polymer solution or polymer melt through a nozzle, maintained at a sufficiently high temperature, which makes it possible obtaining polymer nanofibers, rods, needles, spheroids, and spheres [32,33]. It is worth mentioning that the electrohydrodynamic jetting yielding fibers is usually called electrospinning. In electrohydrodynamic jetting, one electrode is a nozzle through which a thin stream of polymer solution or polymer melt is discharged. The other electrode is a plate called a collector. The stream of solution or polymer melt moving between the electrodes is solidified by evaporation of the solvent or cooling of the polymer melt.

In 2012, D. Crespy et al. showed how electrospinning can be adapted to produce polystyrene microspheroids [32]. In brief, polystyrene microspheres suspended in an aqueous-acetone PVA solution were first swollen with toluene, and then the suspension was used as a substrate for electrospinning. During this process, the events presented below occurred almost at the same time. The stream leaving the nozzle was accelerated in the electric field between the nozzle and the collector, which elongated it and reduced its cross-section. As a result, polystyrene particles plasticized with toluene were subjected to compressive and tensile forces, which transformed them into microspheroids. Adjusting the voltage and distance between the nozzle and the collector controlled the aspect ratio of the spheroidal particles. At the end of the spinning process, solid PVA nanofibers containing polystyrene microspheroids were collected on the collector plate. Finally, the polystyrene spheroids were isolated and purified in the same way as the previously described microspheroids produced by stretching PVA film loaded with microspheres.

The electrospinning method yielded polymicrospheroids with AR in the range of 1.5 to 2.4. The advantage of manufacturing microspheroids by electrospinning lies in its simplicity. Preparation of the PVA matrix with embedded dispersed microspheres and their conversion into microspheroids takes place in a single stage. Moreover, electrospinning is a continuous process enabling the fabrication of a large number of microspheroids. The disadvantage is a narrow range of aspect ratios in which the microspheroids were produced.

Proper adjustment of the polymer concentration and flow rate in the polymer solution jet enabled the direct production of polymer spheroids by electrohydrodynamic jetting [33]. However, the product also contained an admixture of particles with other shapes [33]. It is worth noting that bi- and tri-component particles (e.g., containing poly(lactic acid)-b-poly(glycolic acid) with different proportions of the copolymer block lengths) were produced by using nozzles with side-by-side arranged two or three outlets supplying different polymer solutions [33].

### 2.6. Preparation of Spheroid-like Particles Obtained by Adsorption of Amphiphilic Copolymers on Solid Particles and by Copolymer Self-Organization Processes

**Figure 6:**
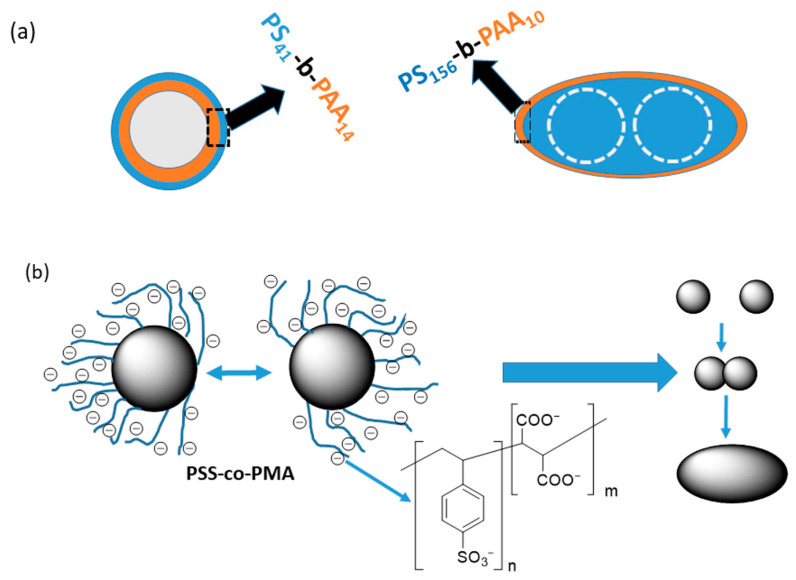
Selected methods for obtaining spheroids: (**a**) formation of spheroidal binuclear particles by adsorption of PS_156_-b-PAA_10_ copolymer with long polystyrene block (blue color) and short poly(acrylic acid) block on the surface of two hydrophobized SiO_2_ microspheres (based on data from ref. [34]); (**b**) by dimerization of spherical polystyrene particles containing PSS-co-PMA polyelectrolyte on the surface (reprinted with permission from the ref. [35]).

Silica (SiO_2_) can be obtained in the form of particles of various shapes and sizes, ranging from nano- to micrometers. Due to the presence of -OH groups on the surface, silica can be functionalized with alkoxysilanes. For example, the reaction of n-octadecyltrimethoxysilane with the aforementioned hydrophilic silica transformed it into hydrophobic particles that effectively adsorb hydrophobic substances. Using this method, Hamada et al. obtained spheroidal particles by adsorbing the amphiphilic di-block polystyrene-b-poly(acrylic acid) (PS-b-PAA) copolymers on the surface of the hydrophobic silica microspheres [34]. The copolymers were synthesized using the tellurium-mediated radical copolymerization (TERP). A series of copolymers with polystyrene blocks containing 41, 86, and 156 styrene constitutional units and poly(acrylic acid) blocks with 14, 13, and 10 constitutional units was prepared. In the next step, the hydrophobized SiO_2_ microspheres, with diameters ranging from 70 to 130 nm, and the chosen copolymer samples were mixed in dimethylformamide. Then, water was added to the suspension of microspheres in the polymer solution. During the above-mentioned process, the polystyrene blocks are adsorbed onto the surface of the hydrophobic silica in a way that exposes the hydrophilic poly(acrylic acid) blocks to the rich-in-water continuous phase. As a result, the core–shell particles, schematically shown in Figure 6a, are formed. The authors found that for the more hydrophobic silica, the copolymer layer was thicker [34].

It is worth noting that in the case of PS-*b*-PAA with the longest polystyrene block (DP = 156), the formation of a fraction of spheroid-like particles with two or more silica nuclei was observed [34]. The very long polystyrene blocks bind to more than one hydrophobic silica nucleus.

The method illustrated in Figure 6a enables the synthesis of spheroids with an aspect ratio close to 2 and a long axis length ranging from 200 to 300 nm. However, it requires further elaboration. Currently, in addition to spheroids, the products contain too large fractions of particles of various other shapes.

### 2.7. Microfluidic Methods

The microfluidic method for obtaining polymer microparticles is unique. It allows all steps, from monomer to microparticles with well-controlled shape, size, and narrow size distribution, to be carried out continuously in a single device [17,35]. The device consists of two parts. The first is used to prepare droplets of monomer and photoinitiator solution dispersed in a continuous phase containing an emulsion stabilizer. The second is the reactor, where the polymerization takes place. The first part consists of two coaxial capillary channels connected to a nozzle. The inner one contains a monomer and photoinitiator solution. A fluid with dissolved surfactant is pumped through the outer capillary. The liquids in the inner and outer capillaries must be immiscible. As a result, the liquid at the outlet from the nozzle contains droplets of monomer and photoinitiator solution dispersed in a liquid supplied from the outer capillary. Such a microfluidic flow-focusing device (MFFD) can produce from 100 to 1000 droplets per second [17]. Then, the aforementioned suspension flows through the second part, where photopolymerization takes place. Polymerization converts monomer droplets to polymer particles. The process is continuous. A similar device can be used for the synthesis of particles by thermal polymerization. It is important to maintain polymer particle suspension at a temperature above the temperature of polymer gelation, at which the particles are moldable. The particles obtain their final shape in the outlet channel from the reactor. Outside of the reactor, the suspension is quickly cooled, and the particles retain their shape.

The shape of the particles depends on the diameter of the initial monomer droplets and the geometry of the outlet channel. The outlet channels had a square or rectangular cross-section. When the diameters of the formed particles are smaller than the height and width of the channel, the flow of particles is unimpeded, and their dimensions do not change. In this case, spherical particles are produced. When the cross-section of the outlet channel is square, and the distance between opposite walls is smaller than the diameter of the moldable polymer particles, the particles are elongated as they pass through the outlet channel. When the difference is small, the final particles resemble slightly elongated spheroid-like spheres. However, when the aforementioned difference is three or more times greater, the particles produced are rod-shaped with rounded ends. The last type of microfluidic flow focusing device discussed had a rectangular outlet channel with a larger inner width than the inner height. After optimizing the flow rate, the device was used to produce polymer particles using tripropyleneglycol diacrylate, dimethacrylate oxypropyldimethylsiloxane, divinylbenzene, ethyleneglycol diacrylate, and pentaerythritol triacrylate mixed with 1-hydroxycyclohexylphenyl ketone as a photoinitiator in aqueous suspensions containing sodium dodecyl sulfate [17]. Using an outlet channel with various geometries, rods, flattened disks, and prolate spheroids were obtained. It is worth noting that there is enough space on a 2 × 3-inch substrate to mount the 10 basic units presented earlier. Such an assembly can produce between 10^6^ and 10^8^ particles per hour.

The literature contains descriptions of several types of microfluidic devices for the synthesis of non-spherical polymeric particles. However, regardless of the specific design concept, all of them include an element that produces the emulsion of the monomer(s), an initiator and stabilizers for the emulsion, a reactor that converts the monomer droplets into spherical polymer particles, and an element that transforms the spherical particles into non-spherical particles or particle aggregates. For example, Visaveliya et al. constructed a device in which an emulsion was produced in a reactor with a micro-hole plate, and non-spherical particles were produced not by the deformation of spherical particles, but by their controlled aggregation and fusion [35].

The process yielded poly(methyl methacrylate) particles cross-linked with ethylene glycol dimethacrylate containing adsorbed polyelectrolytes, the sodium salt of poly(4-styrenesulfonic-co-maleic) acid, sodium poly(p-styrene sulfonate), and the sodium salt of polyanetholesulfonic acid. The diameters of the primary spherical polymer particles depended on the diameters of the holes in the plate reactor. The diameters of the holes in the plates used by Visaveliya were 5, 20, and 40 μm [35]. The shapes of the resulting particles depended on the structure and concentration of the polyelectrolyte, as well as the flow rate. Polymerization carried out in the presence of polyelectrolytes leads to the formation of a so-called “soft” electrical layer. Such a layer enables the polarizability of the resulting microspheres, and ultimately, as a result of less repulsion, particles can approach each other at close distances, as shown in Figure 6b. For example, using a sodium salt of poly(4-styrenesulfonic-co-maleic acid) and optimizing the polyelectrolyte concentration, a product with a 70% spheroid fraction was obtained.

### 2.8. Deformation of the Spherical Particles to Spheroids by Irradiation

Section 2.1 discusses the transformation of spherical particles into spheroids as a result of external forces of interaction of the spheres with the matrix during matrix elongation. This section describes the preparation of spheroidal particles from spherical particles made of polymers with photosensitive ligands. The particles were stretched by internal mechanical forces resulting from irradiation of the particles with planar polarized UV laser light. In the study, microspheres with diameters in the range of 160–310 nm were obtained by self-organization of amphiphilic copolymers with azo groups in the side chains (-C_6_H_4_-N=N-C_6_H_4_-COOH). The research showed that these particles irradiated with an argon laser at a wavelength of 488 nm and a power density of 150 mW/cm^2^ underwent elongation in the plane of light polarization [36]. It was found that the degree of particle deformation increased with increasing density of azochromophores in epoxy polymers and with increasing irradiation time of polymer microspheres.

Li et al. speculate that the change in particle shape occurring below the T_g_ temperature of the polymer is caused by the absorption of light by chromophore groups properly oriented with respect to the plane of light polarization and a configuration change from trans to cis in the -N=N- groups [37]. Such a configuration change has been observed previously [38]. The transition from trans to cis causes a large deformation in a direction that depends on the orientation of the plane of light polarization, resulting in elongation of the molecule. However, it was found that when the deformed particles were heated above the T_g_ of the polymer/copolymer, the elongated spheroids returned to spherical particles [39].

### 2.9. Spheroidal Particles Processes

Block copolymers built from individual polymer segments with significantly different properties exhibit phase microseparation. Linear block copolymers can be obtained through controlled polymerization methods, such as ring-opening metathesis polymerization (ROMP) [40]. In thin films formed by block copolymers, phase microseparation can lead to a layered structure with an orientation parallel or perpendicular to the substrate [41]. This phenomenon can also result from the different affinity of the individual blocks to the environment in which the polymer is located. As a result, polymer particles can be obtained with anisotropic internal and layered structures, such as concentric spheres (bulbs) or striated structures [42]. One method of obtaining polymer nano- and microspheres is to evaporate the solvent from an emulsified polymer solution in water in the presence of a surfactant (CTAB—cetyltrimethylammonium bromide).

Surfactants have an affinity for both the environment and the polymer, or in the case of block copolymers, for a particular block of the copolymer. After evaporation of the solvent from the dispersed phase, which is a solution of the block copolymer containing the surfactant, the dispersion of polymer particles is obtained by phase microseparation. If we designate the diblock copolymer as PA-b-PB, depending on which copolymer block has a higher affinity to surfactant, the microspheres with a normal or inverted phase arrangement are obtained. For one surfactant, the nucleus is the PA block, and the outer layer is the PB block. For another surfactant with the opposite affinity, the PB block is the nucleus, and the outer layer is the PA block, respectively.

**Figure 7:**
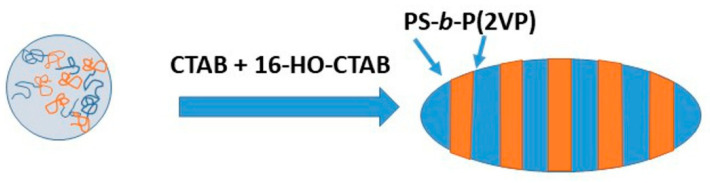
Spheroids obtained by mixed surfactant strategy for controlling the self-assembly of symmetric PS-b-P2VP by solvent evaporation from droplets. CTAB denotes N,N,N-trimethylhexadecane-1-ammonium bromide. Reprinted with permission from the ref. [43].

The ability to reverse the sequence of microsphere layers suggests that in a mixture of surfactants with affinity for different blocks, it is possible to obtain environmentally neutral layers and, consequently, form parallel layers similar to thin films. When the system contains only one surfactant, the extreme structures are particles with a “light bulb” shape, with reversed phase order. In surfactant mixtures, intermediate forms between “bulbs” are observed; cones with a spherical basis and spheroids, in both cases with a layered structure, are obtained. In this way, Klinger et al. obtained spheroidal particles consisting of transverse, alternating layers of polystyrene (PS) and poly(2-vinylpyridine) (P2VP), as is shown in Figure 7 [43]. The authors found that N,N,N-trimethylhexadecane-1-ammonium bromide (CTAB) interacts selectively with polystyrene, while its hydroxyl derivative (16-HO-CTAB) interacts with poly(2-vinylpyridine). Taking advantage of this fact, the researchers used a mixture of surfactants in an emulsion, in which the dispersed phase was a solution of the copolymer in chloroform. After evaporation of the chloroform, spheroidal particles were obtained.

The authors found that the spheroid shape parameter depends on two factors. The first is the molar mass of the individual blocks. Using block copolymers with *M*_n_ of PS and P(2VP) blocks, respectively: 20–20 kg/mol, 41.5–41 kg/mol, 102–97 kg/mol, they found that the greater the length of an individual block, the more elongated spheroids were formed—the aspect ratio was in the range of 1.05–1.50 for the lowest molecular weight copolymer, while an aspect ratio between 1.5 and 2.3 was determined for the highest molecular weight copolymer. The second factor influencing the degree of particle elongation is the original size of the particle from which the spheroid is formed; correspondingly, smaller shape factor values were obtained for smaller particles and larger ones for larger particles. Finally, the authors obtained particles with sizes ranging from 100 to 1000 nm.

Due to the copolymers used, the particles are sensitive to acidic environments. Specifically, uncrosslinked P2VP domains undergo protonation and disentanglement in acidic environments, resulting in the ellipsoidal particles disintegrating into polystyrene disks surrounded by a crown of P2VP polycations. In contrast, cross-linked P2VP domains reversibly swell in acidic medium and shrink in basic medium, but the particles do not disintegrate into disks.

Block copolymers PS9.8 k-b-P(4VP)10 k (where subscripts indicate the average molar mass of each block) were also used by Deng et al., who produced particles with a size similar to those discussed previously [43]. It has been observed that spheroid formation due to the emulsification of the chloroform copolymer solution in water is enhanced by the presence of PVA in the aqueous phase. Removing PVA and allowing solvent vapors to diffuse deeply into the suspension of spheroids in water causes the spheroids to swell and start chain relaxation processes. This relaxation changes the shape of the spheroids into spherical particles with a layered morphology, alternating between PS and P(4VP). Spheroids can be reformed by adding PVA to the system and removing the organic solvent. The resulting particles are cone-shaped with a spherical base. Notably, this process is completely reversible [43].

**Figure 8:**
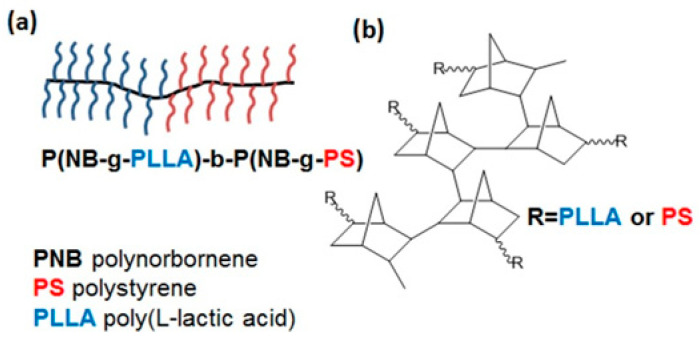
Architecture of (P(NB-g-PLLA)-*b*-P(NB-g-PS)) bottlebrush block copolymer described in ref. [44] (**a**). Chemical structure of the polynorbornene backbone (**b**). Reprinted with permission from the ref. [44].

Particles with analogous morphology were obtained using block copolymers with brush architecture. The blocks were polynorbornene chains, where one block was grafted with polystyrene side chains (PNB-*g*-PS) and the other with polylactide side chains (PNB-*g*-PL), as shown in Figure 8a,b [44].

In the first step of copolymer synthesis, polystyrene (*M*_n_ = 2500 g/mol, *M*_w_/*M*_n_ = 1.08) and polylactide (*M*_n_ = 2800 g/mol, *M*_w_/*M*_n_ = 1.17) were synthesized and functionalized with terminal norbornene groups capable of ring-opening polymerization according to the mechanism of metathesis (ROMP). These oligomers constituted macromonomers in the subsequent synthesis of a series of block copolymers (P(NB-g-PS)-b-P(NB-g-PLLA)) by sequential addition of macromonomers with appropriate block lengths (theoretically:) 50–50, 100–100, and 150–150 constitutional units. The dispersion coefficient of the obtained copolymers was in the range *M*_w_/*M*_n_ = 1.02–1.06.

Colloidal particles were obtained by evaporation of benzene from the emulsion of the copolymer solution in the presence of CTAB. The authors found that the highest molar mass of the copolymer promoted the formation of layered spheroids, as shown by the PS-b-P2VP particles in Figure 7. This behavior can be explained by the tension of the main chain, which increases with increasing chain length when spheroidal particles are formed. In this situation, it is more favorable to form spheroidal particles with a morphology of alternating striations. As in the previously cited work [43], the results showed that the larger the diameter of the starting particle, the higher the aspect ratio of the obtained spheroids, which ranged from 1.5 to 2.8. The spheroidal particles with volumes in the range of 5 × 10^6^–1 × 10^9^ nm^3^ were obtained. The method is limited by the following factors: the inability to obtain particles with core–shell morphology and insufficient control of the size and aspect ratio of the obtained particles.

### 2.10. Overview of the Methods of Obtaining Polymer Prolate Spheroids

An overview of methods leading to polymer prolate spheroidal particles is included in Table 1.

## 3. Phenomena Accompanying the Self-Organization of the Particles at the Phase Boundaries

Particles with sizes ranging from tens of nanometers to tens of microns are model objects for studying phenomena that occur at phase boundaries. They can be components of monolayers or multilayers of particles with a high degree of spatial order, which are useful for constructing biosensors [45], materials for optoelectronics [46], or emulsion/foam stabilizers [47]. Polymeric particles of various shapes, morphologies, and sizes down to about a few microns can adsorb at phase boundaries:

- liquid–liquid and liquid–gas;
- liquid-solid;
- solid–liquid–gas,

whereby in most studies, the gas is air.

The beginning of research on such systems and phenomena of adsorption of solid particles at phase boundaries can be taken in 1903–1907, when Ramsden and Pickering described emulsions stabilized by so-called insoluble emulsifiers, such as colloidal Ca(OH)_2_ particles, which fixed the emulsion of paraffin in water. Emulsions of this type are called Ramsden-Pickering emulsions [48,49]. More intensive research on these types of systems began in the second half of the 20th century, mainly due to the development of nanotechnology as well as the advancement of the methods for obtaining nano- and microparticles.

Particles adsorbed at the phase boundary are different from the particles inside the dispersing phase and are characterized by parameters that are not relevant in the case of particles in dispersion. Issues that researchers deal with in the case of systems of this type include the equilibrium position of particles at the phase boundary, capillary interactions, particle organization, shear and compressive forces in the monolayer, and applications of surfaces coated with colloidal particles. The research relating to these topics has been described, including works by Böker et al. [50], Botto et al. [51], and Anjali et al. [52]. The authors proposed methods for the study of the particles’ behavior at the phase boundaries and introduced appropriate terminology for anisotropic particles.

Adsorption of colloidal particles at the interface plays a key role in stabilizing phase boundaries in emulsions, foams, gels, and other materials with highly developed contact surfaces [53,54]. Like low-molecular-weight surfactants, particles (mainly polymeric) stabilize a system when they have affinity for both phases, allowing them to adsorb at the interface.

Adsorption of colloidal particles at the liquid–liquid or liquid-air interface results in the formation of two new phase boundaries, at the expense of reducing the area of the original contact surface. The degree of reduction in the interfacial area between two liquids depends on several factors: the size and shape of the particles, the angle of wetting of the particles by the two phases (CA), and the degree of deformation of the phase boundary around the particle. However, not in every case do particles easily adsorb at the phase boundary—in some cases, they may remain dispersed in one liquid. For a particle to adsorb at the phase boundary, it cannot be exclusively hydrophilic (in the case of the aqueous phase, the wetting angle is zero) or exclusively hydrophobic (180° in the case of water). When particles anchor at the water/air interface, a change in surface tension is observed. Particles with no charge anchor most effectively, and in the case of particles with a surface electric charge, the most effective adsorption at the boundary occurs under isoelectric point conditions, where the particles are electrically inert—then they do not interact repulsively with particles remaining in the liquid and do not impede the adsorption of other particles [52].

**Figure 9:**
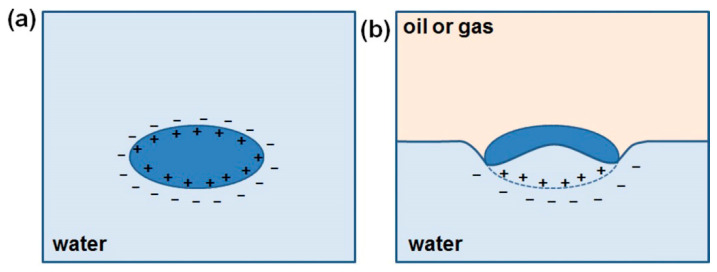
Comparison of charge distribution of the particle (**a**) in water, and (**b**) at the phase boundary.

Spherical and non-spherical particles adsorbed at the boundary show both similarities and differences. Even particles with a chemically homogeneous surface take on a dual character (so-called Janus particles) when one part of the particle is immersed in a liquid and the rest is in contact with air (or gas) or a liquid with a different hydrophilicity. For example, molecules may have functional groups on the surface that dissociate in polar solvents (such as -COOH groups in water) and do not dissociate in an organic solvent that is the second phase (such as -COOH groups in hydrocarbons). A molecule adsorbed at the polar-nonpolar interface has an electric charge only on the part of the surface that is in contact with the polar solvent. For this reason, a particle which has a chemically homogeneous surface in the bulk gains duality when it is at the liquid–liquid interface (see Figure 9).

**Figure 10:**
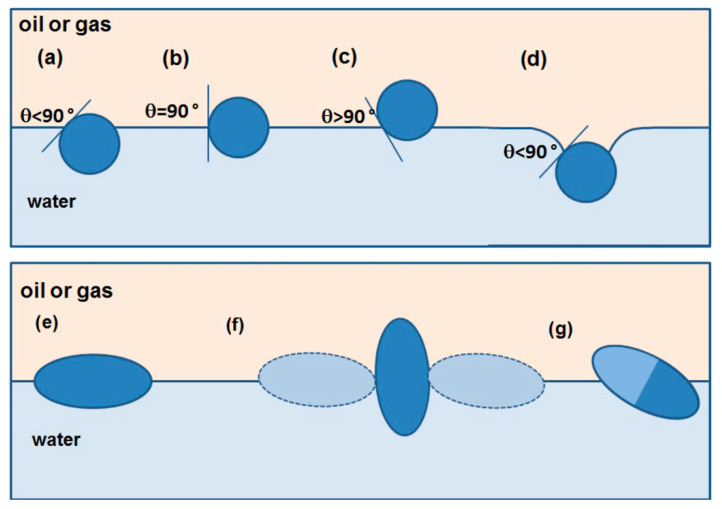
Positions of the particle at the liquid–liquid (or gas) interphase to the wetting contact angle at the phase boundary: (**a**–**c**) equilibrium position of the particle at the liquid–liquid interphase to the wettability of the particle surface layer; (**d**) deflection of the boundary due to the large mass of the particle; (**e**–**g**) positions of the prolate spheroid at the liquid–gas boundary versus the properties of the surface layer. Reprinted with permission from the refs. [52,55].

Based on the review work of Böker, Botto, and Anjali et al., it is known that the equilibrium position of the particles is a measure of the wettability of the particles and is characterized by the wetting angle (denoted as Θ in Figure 10) at the boundary of the three phases, defined by Young’s equation (Equation (9)) [50,51,52]:

(9) $$\cos\theta =\frac{\gamma_{pf1-}\gamma_{pf2}}{\gamma_{f1f2}}$$

where γ*_pf_*_1_ is the surface tension between the particle and the lower density liquid, γ*_pf_*_2_ is the surface tension between the particle and the higher density liquid, and γ_f1f2_ is the surface tension between the two liquids [51,52]. Depending on the hydrophilicity of the surface, the particle occupies a different position at the interface (see Figure 10a–d). A particle adsorbed at the interface reduces the liquid–liquid interface, creating two new liquid-solid interfaces or one liquid-solid and one liquid–gas interface. The energy effect of trapping a particle at the interface is a measure of the stability of the particle in the system. The said energy effect depends on the wetting angle at the interface of the three phases and the particle size. The energy of particle entrapment increases with the square of the particle size and, for particle sizes below 1 µm, reaches values that cause irreversible particle adsorption [51,52].

Spherical particles with a chemically homogeneous surface assume only possible orientations at the phase boundary, and the only parameter that characterizes them is the depth of immersion in both phases, as shown in Figure 10a–d. For spheroidal particles, on the other hand, different particle orientations at the phase boundary are additionally possible, as shown in Figure 10e–g.

The study showed that for spheroidal particles at the oil/water or air/water interface, the energetically most favorable orientation is lateral (Figure 10e). However, in a special case, an orientation perpendicular to the phase boundary is also possible. Namely, due to the compression of the particle layer at the phase boundary under the influence of lateral pressure (p) exerted on the particle by neighboring particles, there is a “reversal” of the particle, leading to its reorientation (see Figure 10f) [55].

In contrast, the diagonal position is most favorable for particles whose surface can be divided into two domains of different composition (so-called Janus particles), as shown in Figure 10g.

**Figure 11:**
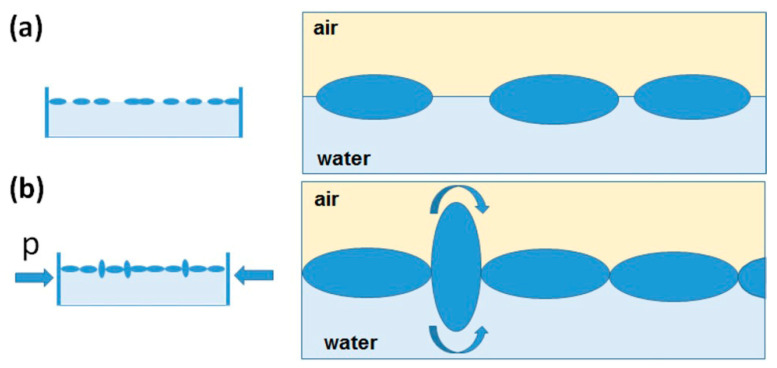
Behavior of the prolate spheroids monolayer at the water/air interface upon lateral pressure (p) of the surface area (side view): (**a**) before compression; (**b**) after compression, using Langmuir trough. Reprinted with permission from the ref. [55].

The results showed that “flipping” occurs especially in the area of highest particle packing at the interface due to surface compression. Further compression results in the formation of multilayers of particles that remain fragmented even after surface expansion [55]. The behavior of spheroidal particles during compression at the interphase is shown schematically in Figure 11a,b.

The deformation of the phase boundary is caused not only by the relatively large mass of the particles. An additional factor at the phase boundary is capillary phenomena. Deformation does not occur in the case of particles with low mass, additionally isotropic in shape, morphology, and surface chemistry. In other cases, changes in the level of the phase boundary associated with capillary phenomena are observed. Even spherical particles can cause deformation of the liquid–liquid interface if their surface is not chemically homogeneous or is inhomogeneous due to surface properties. Anisotropic particles at the liquid–liquid interface are so-called monopoles, dipoles, quadrupoles, or, more generally, capillary multipoles.

**Figure 12:**
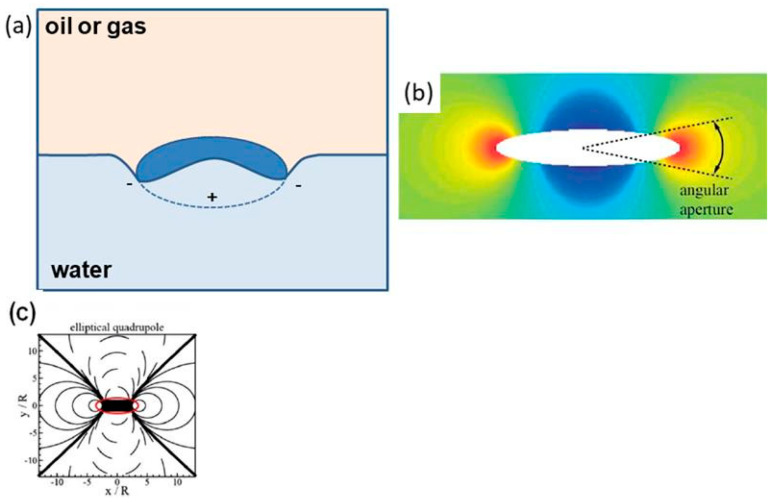
Deformation of the phase boundary caused by a spheroidal particle being a capillary quadrupole: (**a**) side view; (**b**) top view; (**c**) iso-height contour obtained from simulation of an elliptic quadrupole. The blue and red regions indicate downward and upward deflections, respectively. Reprinted with permission from the ref. [50,51].

Capillary effects are positive and negative, depending on whether the liquid–liquid interface near the particle is raised or lowered. Figure 10d shows a spherical particle with only a negative capillary effect (capillary monopole) due to the large mass of the particle. Particles with the shape of elongated spheroids are capillary quadrupoles, where two poles with positive effect and two with negative effect are observed. A side view of the phase boundary deflection caused by a spheroidal particle with a contact angle < 90° with the water surface is shown in Figure 12b,c [51,52].

**Figure 13:**
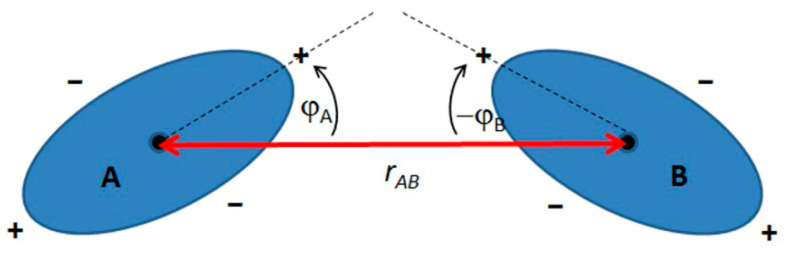
Reciprocal interactions of two spheroidal particles. Reprinted with permission from the ref. [51].

Monopoles, dipoles, and capillary multi-fields, like their electric or magnetic counterparts, interact repulsively or attractively, but with the difference that unipolar effects are responsible for the attractive interactions, and differential effects are responsible for the repulsive ones. Thanks to this phenomenon, particles with identical interactions at the phase boundary (e.g., air bubbles on the surface of a liquid) tend to cluster. In the case of spheroidal particles, this means that the particles will interact attractively side to side or end to end, and repulsively, side to end. A diagram of the interactions of two spheroidal particles is presented in Figure 13.

Equation (10) represents the expression proposed by Stamou et al. [56]. For the case of the interaction of two spheroidal particles described above:

E_AB_ = −γ (12πH_p_^2^ r_p_^4^)/(r_AB_^4^) cos(2φ_A_ + 2φ_B_) (10)

where E_AB_ stands for the energy with which particles A and B interact, γ denotes the surface tension at the liquid–liquid interface, H_p_ is the amplitude of the deformation of the interface, r_p_ is the radius of the contour around which the deformation of the interface occurs, and φ_A_, φ_B_ are the “angles of bond”. In turn, r_AB_ is the “bond length”, which is the distance from the center of one particle to the center of the other.

Equation (10) shows that the largest energy gain of the system occurs when the particles are parallel to each other; moreover, lateral interactions are stronger than end-to-end interactions.

It was also found that particles with an aspect ratio of less than 1.5 behave similarly to spherical particles, whereas more elongated particles exhibit different properties due to their shape [56]. The authors also report that capillary interactions between particles are much stronger for elongated spheroids than for spherical particles [57].

**Figure 14:**
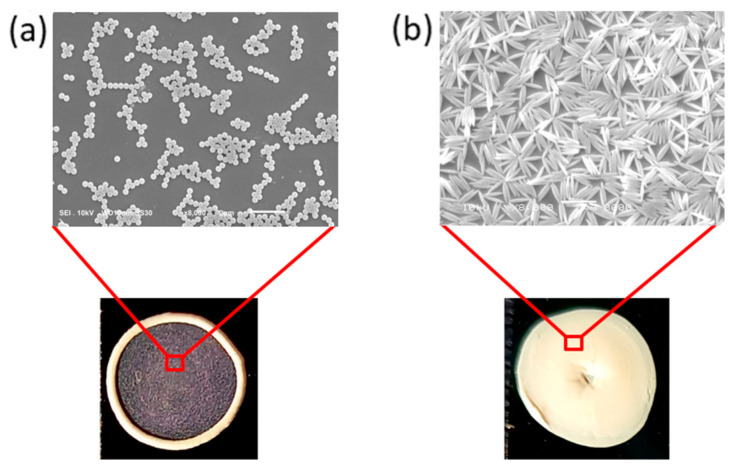
SEM image and digital camera image of a droplet of the aqueous particles’ suspension deposited on the glass plate after drying: (**a**) microspheres; (**b**) prolate spheroids. Reprinted with permission from ref. [59].

In addition, the behavior of spherical and spheroidal carrier phase particles from a suspension of particle droplets during evaporation was compared [57]. The study used aqueous suspensions of polystyrene spheroids with an aspect ratio in the range of 1.05–3.50. The spheroids were obtained from polystyrene microspheres with an average diameter of 1.3 µm by thermomechanical deformation of the microspheres. It was found that only spheroidal particles adsorb at the liquid-air interface, while spherical particles remain exclusively in the liquid. Moreover, the study showed that after depositing a droplet of spheroid suspension on a solid substrate, spheroidal particles occupy the entire interfacial region, deforming the water–air interface. It was observed that water is pulled up in the center of the spheroidal particle and pushed down near the ends. This behavior results in local interface distortion. To minimize the interface distortion, surface tension forces push the particles together. Consequently, strong lateral capillary forces lead to uniform particle coverage at the water–air interface. For spherical particles at the water–air interface, the situation is different, as the spheres do not distort the interfacial region and accumulate near the droplet edge. As a result of stronger interactions and greater deformation of the water–air interface by elongated spheroidal particles, the mobility of anchored particles is significantly reduced. In the case of spherical particles, there is an accumulation of particles at the edge of the droplet, resulting in the formation of a characteristic ring, the so-called “coffee ring,” as shown in Figure 14a [58]. In contrast, in the case of spheroidal particles, the coffee ring effect is much smaller or completely inhibited (see Figure 14b). Elongated particles with an aspect ratio greater than or equal to 1.5 are evenly distributed throughout the area occupied by the droplet.

The larger specific surface area at the curved edges of the droplet on a solid substrate causes the liquid in the droplet to flow toward the droplet’s edge. When the liquid is a suspension of microspheres or microspheroids, the flow of liquid moves particles that are far from the absorbing support, where the adsorbed particles are much less mobile. The situation is more complex when the particles and carriers are electrically charged, and their interactions are modified by an electric field [52]. It is also clear that the adsorption of the particles from a dense suspension during drying strongly depends on the shape of the particles (see Figure 14).

## 4. Examples of the Self-Assembly of Prolate Spheroids

The formation of regularly arranged colloidal particles is attracting the attention of researchers involved in fundamental studies of the disorder-to-order transition. Two-dimensional particle assemblies also have practical implications for the design of nano- and microelectronic components in miniature lab-on-chip devices. The most promising and relatively inexpensive technologies are those based on particle self-organization, also known as particle self-assembly. The term “self-organization” is used in many fields of science, including physics, physical chemistry, chemistry, materials science, biology, and even sociology. In some of these fields, specific modifications to the meaning of the term “self-organization” have been introduced. In this study, we use the term following its common usage in colloid science. In colloid science, self-assembly means a spontaneous process driven by local interactions. As a result, the disordered particles form ordered structures. The conditions of the process are usually prepared by the researcher, but the process itself begins and proceeds without his intervention.

The literature contains descriptions of the formation of ordered particle systems, called colloidal photonic crystals. In most cases, the colloidal crystals involve spherical particles. A comprehensive review of the preparation strategies and properties of ordered systems obtained with or without the action of external fields from spherical, Janus-type, plate-like, tetrahedral, and rod-like particles is presented in ref. [60]. The review highlights the influence of the shape and morphology of the building blocks on self-organization behavior. These systems can form monolayers (2D) and multilayers (3D). Two-dimensional systems can be formed on the surface of a solid and can also be adsorbed at the liquid–liquid interface, e.g., water-oil. In turn, polymer particles can be adsorbed on a flat surface or colloidal particles with opposite charge [61,62]. The latter suspensions of particles stabilized with solid colloids are so-called Pickering emulsions [63].

Lewandowski et al. described an emulsion of water in hexadecane, stabilized by cylindrical particles obtained from epoxy resin [64]. These particles formed a monolayer with an ordered structure at the water-hexadecane interface, i.e., a two-dimensional colloidal crystal. Basavaraj et al. investigated the formation ordered assemblies of spherical or spheroidal particles at the non-miscible liquid–liquid interface [65].

Spheroidal polystyrene particles were synthesized using the classical method discussed in Section 2.1 of this work. This method involves the synthesis of spherical particles by emulsion polymerization, followed by their elongation in a PVA matrix. After isolation and purification, the particles were used in further studies. Electrically charged spherical polystyrene particles were synthesized by potassium persulfate-initiated polymerization, generating polymers with sulfate anion end groups. The diameter of particles was equal to 3.1 µm, and a relatively high (negative) zeta potential ζ = −57 ± 3 mV was noticed (measured in water at 25 °C). On a flat surface, the spherical particles formed two-dimensional hexagonal structures. In the next stage, the authors obtained spheroidal particles from the above-mentioned microspheres with an aspect ratio of 2.55 and 5.30, respectively. Polystyrene particles with negligible surface charge were synthesized in a polymerization initiated by nonionic AIBN (azobis-(isobutyronitrile)). Their average diameter was 2.8 µm. These particles were transformed into spheroids with AR = 5.50. The availability of these spheroids made it possible to verify the role of electric charge in the distribution of particles. As a result, it was found that uncharged spheroids initially formed loose chain structures at the water–decane interface, with particles arranged end-to-end. These systems evolved over the first 30 h by increasing the packing density of the particles. Contact between particles changed from end-to-end to side-to-side. For particles with a significantly higher surface charge, it was found that the ordering of particles depends on the type of phase boundary. At the water–air interface, the particles mainly formed triangular structures. At the water–decane phase boundary, it was observed that the spheroidal particles were initially dispersed like microspheres and then joined together in chains through interactions between their ends. These structures underwent further changes during the first 30 h, and the particles arranged themselves sideways.

One method of obtaining ordered arrangements of multilayered particles is to evaporate the scattering phase from a drop of the concentrated particle suspension applied to the surface of a solid [57,58]. As mentioned earlier, in the case of suspensions of spherical particles, the accumulation and formation of ordered arrangements in the form of a ring near the edge of the droplet (the “coffee ring” effect, visible in Figure 12a) is observed. Spheroidal particles have a much lower tendency to accumulate at the edge of the droplet. In the case of overly diluted suspensions of spheroidal particles, instead of orderly arrangements, uniformly distributed particles are observed across the entire surface of the solid with which the droplet was in contact [56].

In the case of suspensions of particles with high concentrations, ordered multilayers are obtained over the entire area of the solidified droplet. Due to the much smaller tendency of spheroidal particles to accumulate at the edge of the droplet than is the case with spherical particles, multilayers of spheroidal particles are characterized by much smaller differences in thickness between the central and the outer part of the multilayer. The “coffee ring” effect formed by the spherical particles is related to faster evaporation of the liquid at the edge of the droplet, which has to do with the greater curvature of the droplet and thus the greater development of its surface area at the edge. The results of Das et al. showed that the formation of a “coffee ring” can be suppressed by increasing the curvature of the droplet [66]. In the experiments, “the ring” effect was observed when a droplet of a suspension of spherical particles was deposited on a hydrophilic surface. In contrast, in the case of the hydrophobic surface, the droplet had a much more spherical shape than on the hydrophilic support. Thus, ordered multilayers of spherical particles were obtained over the entire surface occupied by the applied droplet [66].

It might seem that the easiest way to produce regular arrangements of colloidal particles would be to deposit a droplet of a particle suspension in water on a hydrophilic solid support and evaporate the aqueous phase. However, this approach often does not yield good results. As demonstrated in Section 3 of this paper, the difference between the combination of interparticle capillary forces and surface tension at the three-phase droplet boundary is significantly different for spherical and spheroidal particles. As a result, spherical particles accumulate at the edge of the droplet (forming a so-called “coffee ring”), and spheroidal particles adsorb flat across the entire droplet-substrate interface. Figure 14 illustrates this phenomenon. It is worth noting that the “coffee ring” problem was solved by the replacement of the free evaporation of the suspending phase with evaporation during the sample spin coating [67].

In the process mentioned above, the drop of the polystyrene nanosphere suspension in methanol containing Triton X-100 was placed on the 2 × 2 cm^2^ wafer. The spinning consisted of three steps. During the first step, the sample was spun for 10 s at 400 rpm, spreading the suspension evenly. The second step lasted for 2 min. at a speed of 800 rpm to remove the excess of the particle suspension. Eventually, the final step, lasting 10 s with a very high spinning rate, 1400 rpm, was used to remove some residue of the “coffee ring”. Later, the size of the particles in the two-dimensional monolayers was reduced using the radiofrequency etching (RFE), and the final pattern was obtained using the reactive ion etching (RIE) [67].

The above example shows how radiofrequency etching of a two-dimensional colloidal crystal reduces particle dimensions and increases the distance between them. However, a few years later, Isa et al. developed a method that required only simple, homemade equipment to produce regular microsphere arrays with long interparticle distances [68]. The arrays were prepared from positively charged amidine polystyrene particles (polystyrene particles with amidine groups on their surface). The diameters of the particles were in the range from 40 to 500 nm. The arrays were created at the interface between water and hexane in a polypropylene centrifuge tube. Then they were transferred to a silicon substrate (silicon wafers coated with a negatively charged layer of a silicon oxide or silicon nitride) mounted on a rod inclined at an angle of approximately 10° to the horizontal plane. The procedure for creating the particle array and transferring it to the substrate is briefly described below [68]. First, water is poured into the tube. Then, a rod with the substrate is placed in the tube (the substrate is at the bottom). Next, 10 mL of hexane is poured onto the water’s surface, creating a phase boundary between water and hexane. The required amount of particles suspended in a mixture of water and isopropanol is injected into this phase boundary. Once equilibrium is reached, the substrate is removed, collecting the particle arrangement as the substrate crosses the water-hexane interface. The particles adhere to the substrate through electrostatic interactions and are irreversibly adsorbed after drying. The distances between particles can be controlled in the range of three to ten particles. These structures were later used to build biosensors.

There are examples of ordered mono and multilayers of spherical and spheroidal polymer particles obtained by the action of external forces. A good example is the compression of the loose particle assemblies at the liquid-air interfaces.

About a century ago, Irving Langmuir (winner of the 1932 Nobel Prize for his achievements and investigations in surface chemistry) began systematic research on molecular monolayers on the surface of liquids. Together with Katharine Burr Blodgett, they developed a method for sequentially transferring molecular monolayers onto solid substrates, enabling the production of surfaces covered with single and multi-layer molecular assemblies (LB technique). Currently, equipment for such research is available from various suppliers under the name “Langmuir-Blodgett trough.” It was most often used to prepare and study monolayers and multilayers of lipophilic molecules. In the last decade of the 20th century, the LB technique was extended to monolayers of colloidal spherical polymer particles [69]. Monolayers were prepared from spherical particles of a copolymer synthesized from styrene, butyl acrylate, and acrylic acid. The particle diameter was 70 nm. The particles were suspended in a chloroform solution containing octadecylamine, stearic acid, and methyl octadecylate, used as spreading or co-spreading agents. The required amount of particle suspension was added to the top of the aqueous subphase. After evaporation of the chloroform, the loose particle structure was compressed using a movable wall and transferred to the substrate by dip coating. It should be noted that the particles in the monolayers were separated by spreading molecules.

It is worth noting that the LB method can be used not only to prepare monolayers of “hard” polymer nanospheres and microspheres, but also “soft” polymer microgel particles [70].

The paper mentioned above presents a synthesis of two types of microgel particles and their behavior at the water–air interface [70]. The first particles were obtained by radical copolymerization of methyl methacrylate and methacrylic acid esterified with a derivative bearing the oxazoline function. The second had a hyperbranched structure of five generations. The first four generations had poly(vinyl ether-g-(polystyrene-g-(poly(vinyl ether-g-polystyrene))) copolymer structure. The last, at the periphery, was composed of the poly(tri(ethylene glycol)vinyl ether) blocks. The authors performed detailed fundamental physical studies of the spherical microgel particle assemblies at the water–air interface. For example, they found that in the monolayer, the most dense packing of particles corresponds to the hexagonally close-packed arrangement (hcp). Moreover, the paper contains results of determinations of the pressure vs. surface area isotherms and of interfacial viscosity, as well as AFM studies of the microgel particle assemblies transferred onto solid supports. Although the applications of the aforementioned systems remain unknown, they might be considered as interesting objects for adsorption on the walls of vessels as supports for cell cultures.

Noteworthy is the article by Basavaraj et al. on monolayers of spheroidal polystyrene particles at the water–air interface [55]. The monolayers were prepared and studied using the LB technique. The author produced spheroidal particles in the two-step process, the basis of which was presented earlier in Section 2.1. In the first step, they synthesized spherical polystyrene particles in the dispersion polymerization initiated with azobisisobutyronitrile. The diameter of these particles was 2.8 ± 0.1 μm, and the small diameter error indicated a narrow diameter dispersity. The particles were slightly negatively charged. However, the researchers did not explain the origin of this charge. During the next step, the spherical particles were converted to spheroids by placing them in the PVA matrix and stretching it in a controlled manner. Aspect ratios of obtained particles were 2.3 ± 0.3, 5.5 ± 0.8, 7.6 ± 1.2, and 9.1 ± 1.4. The particle monolayer was prepared by spreading the particle suspension in a water-isopropanol mixture over water in a Langmuir-Blodgett trough equipped with a mobile wall at the water–air boundary and a sensor for measuring a surface pressure. A special automated optical microscope system was used to visualize the morphology of particle aggregates during the compression and expansion of the particle monolayer. Parallel to optical observations, the isotherms of surface pressure versus surface area, in which the particles were confined, were recorded. For compression experiments, the sequence of events is as follows. At the initial state, just before compression, the particle monolayer was composed of individual spheroids and loose spheroid aggregates. All were randomly oriented in the plane of the water–air interface. At the beginning of compression, the particles (isolated and in aggregates) were pushed together, making new interparticle contacts, which increased the surface pressure. At the percolation threshold, the particle aggregates formed a dense wall-to-wall particle network at very high surface pressure. In the case of spheroids with a high aspect ratio, the particles formed randomly oriented side-to-side domains. Further compression caused the spheroids to flip by changing their orientation from lateral to perpendicular to the water–air interface. This was the beginning of buckling instability, eventually, with the production of a monolayer only consisting of perpendicularly oriented spheroids, and any further compression of the monolayer without particle deformation was not possible. It is worth mentioning that decompression causes cracking of the monolayer, although spheroids in the clusters of the perpendicularly oriented particles do not change their orientation [55].

The LB technique is not the only method based on the use of external forces (in the case of the LB technique, compression) to create single- and multi-layered spherical and spheroidal polymer particles. Multilayer spheroidal particles arranged in quasi-crystalline arrays were also obtained by applying an electric field as an external factor. Shah et al. used negatively charged polystyrene spheroids with an aspect ratio of 4.3 and an electrophoretic potential of −38 ± 12 mV to prepare colloidal quasi-nematic liquid crystals. The process was carried out at 25 °C in a solution of tert-butylammonium chloride in DMSO (with a conductivity of 3.45 mS/cm) [71]. The particle suspension was introduced between two glass plates spaced 1 mm apart. The glass plates were coated with conductive indium tin oxide (ITO). The device design enabled the observation of particles using a confocal microscope. When an electrical voltage (in the range of 1–3 V) was applied to the electrodes, the particles moved towards the anode. The tests showed that at a voltage of approximately 1 V, the particles on the anode were not ordered, and the final degree of surface coverage by the particles was low (<10%). High packing density and good particle arrangement on the anode were observed at a voltage of 1.85 V. In this case, the microparticles formed quasi-nematic colloidal liquid crystals [71].

It is worth noting the very simple methods that do not require any special equipment for the production of single- and multi-layer ordered polymer microspheres, or microspheroids. These methods utilize processes known as spontaneous self-organization of particles. In this context, the term “spontaneous self-organization” may be misleading without a comment suggesting an apparent lack of interaction between particles and the environment influencing the particle organization process. However, this is not true. In fact, self-organizing particles interact with liquid-solid and liquid-solid-air boundaries.

**Figure 15:**
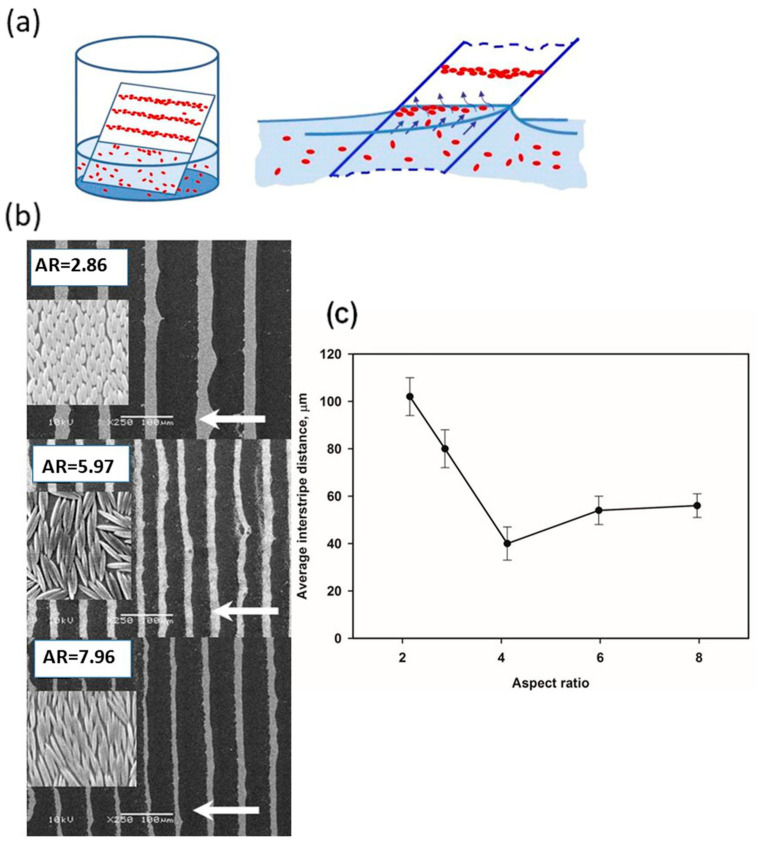
(**a**) drawing illustrating formation of the strip by the prolate spheroids during liquid evaporation; (**b**) SEM microphotographs of stripes and arrangements of the particles in the stripes formed by poly(styrene/polyglycidol) spheroids with AR = 2.15 and AR = 7.96 (authors’ SEM microphotographs [72]); (**c**) dependence of inter-strip distance versus aspect ratio of the microspheroids. Reprinted with permission from the ref. [72].

Furthermore, the systems are open to liquid evaporation, and the particle suspension is subjected to capillary forces at the phase boundaries. The term “spontaneous self-organization” means simply that once started, the process continues without intervention by the researcher. Recently, D. Mickiewicz et al. published the results of their research on the self-organization of spheroidal particles with a polystyrene core and a polyglycidol-rich shell during the dewetting of silicon wafers immersed in 95% ethanol [72]. These spheroidal particles were prepared by stretching microspheres with a polystyrene core and a polyglycidol-rich coating, as described in Section 2.1 and in reference [27,72]. The AR coefficient of the spheroids used for the dewetting experiments ranged from 2.86 to 7.96. The only equipment needed to induce self-assembly of the spheroids is a glass cylinder. The inner diameter of the cylinder and the length and width of the silicon wafer were such that when placed in the cylinder, the wafer was inclined at an angle of 45° to the vertical. The spheroid self-assembly process began after adding a suspension of spheroids in ethanol to the required level and incubating the plate for a specified period of time (usually several days) to evaporate ethanol and deposit the particles. Figure 15a illustrates the spheroid deposition process.

It is worth mentioning that the ethanol meniscus at the ethanol-silicon-air interface was concave, which, due to spatial reasons, caused the spheroids to orient themselves at the liquid-solid-air triple phase boundary along this line. When the ethanol began to evaporate, the meniscus moved down the silicon substrate, pushing the particles and accumulating them. However, when the shear forces exceeded the surface of the ethanol, the meniscus jumped over, starting to accumulate a new batch of spheroids. As the ethanol continued to evaporate, the liquid unveiled the surface of the silicon substrate covered with regularly spaced stripes of spheroids (Figure 15b). The stripes were monolayers of quasi-nematic oriented particles. All stripes were perpendicular to the direction of the meniscus movement. There are also other important characteristics of the stripes. The measurements of the strip width and the distance between stripes depended on the particle aspect ratio (Figure 15c). Both of these parameters were significantly smaller for spheroids with a higher aspect ratio, but the degree of coverage of the silicon substrate was very weakly dependent on AR. This means that the aspect ratio did not affect the number of adsorbed spheroids, but it did affect their distribution on the surface of the silicon wafer.

**Figure 16:**
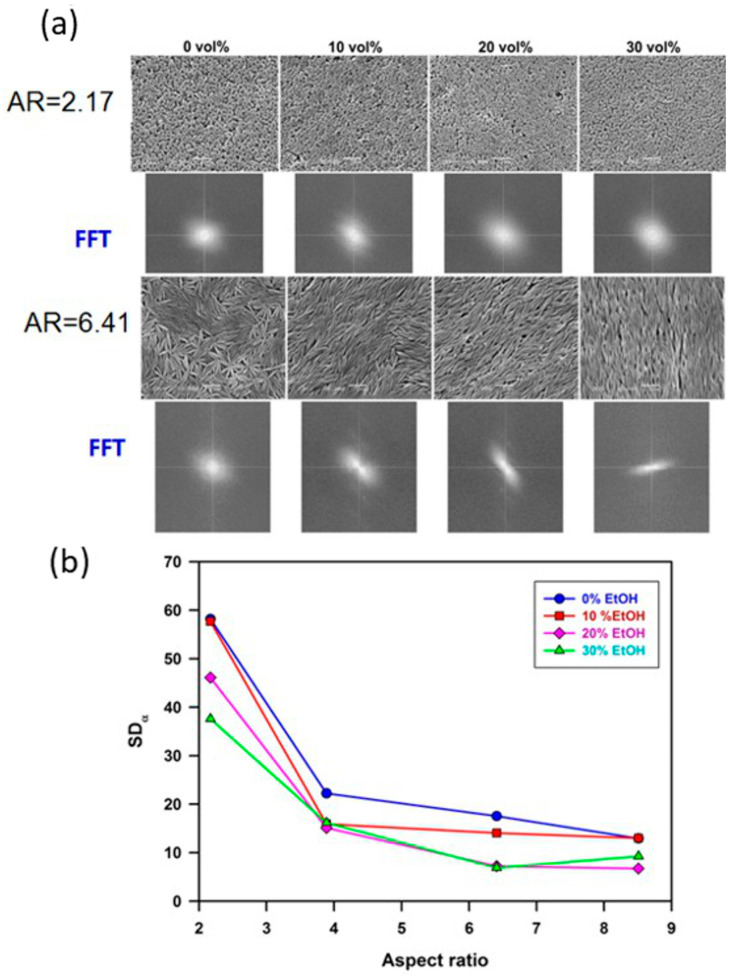
(**a**) exemplary SEM microphotographs of spheroid assemblies, top layers, and FFT images indicating arrangement degree in relation to the addition of ethanol to the particle suspension; (**b**) dependence of standard deviation (SDα) of the particles’ alignment on spheroid aspect ratio. Reprinted with permission from the ref. [58].

The same laboratory also conducted quantitative studies on the formation, morphology, and mechanical properties of 3D colloidal quasi-nematic crystalline assemblies of poly(styrene/polyglycidol) microspheroids [58]. The assemblies were prepared by depositing drops of a suspension of particles with an aspect ratio in the range of 1.0–8.5 on silicon or glass plates and evaporating the liquid phase. This extremely simple process resulted in the formation of micro-particle assemblies with three-dimensional liquid crystal domains of colloid. This material was examined using optical methods, SEM, array fracture, and nanoindentation. As a measure of the quality of spheroid ordering in quasi-nematic domains on the surface of particle arrays, the researchers chose the standard deviation of the angle between any selected reference axis and the direction of the long axis of the spheroid. For a perfectly nematic arrangement of particles, this parameter (SDα) should be 0°, and for a random arrangement of particles, its value should be 90°. Figure 16b shows that for spheroids with AR = 8.5, the SDα parameter ranged from 5° to about 10°, indicating very good alignment. For AR = 2.17, the quality of quasi-nematic ordering was significantly worse (SDα in the range from 28° to 59° depending on the ethanol concentration in the continuous phase of the particles).

In addition, light diffraction studies at the angle of separation from the surface formed by multilayer spheroids provided information on the organization of particles in multilayers. It was shown that the ratio of the interplanar distance between the diffraction planes and the short axis of the spheroidal particles was in the range of 0.6–1.1 for particles with AR in the range of 2.17–8.50, respectively, and depended primarily on the shape factor of the particles, regardless of the ethanol content in the dispersion medium.

Figure 16a also shows 2D fast Fourier transforms (FFT) of SEM images of the upper surface of spheroidal particle assemblies. It is worth mentioning that the shapes of these transforms are fully consistent with the results of the quantitative analysis of the relationship between particle alignment quality and AR. For particles with AR = 2.17, the FFT transformation was isotropic, indicating no directional orientation of particles on the surface of the assemblies, while for particles with AR = 6.41 and 8.50, the FFT transformation was typical for well directionally oriented particle assemblies [58].

**Figure 17:**
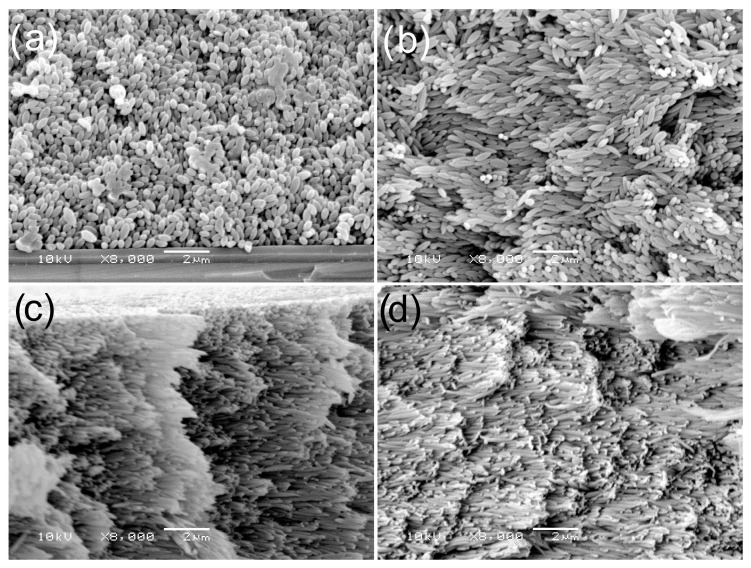
SEM microphotographs of fractures of the P(S/PGL) microspheroid assemblies prepared from particle suspensions in water/ethanol media containing 30% of ethanol. Particle aspect ratio: (**a**) 2.17, (**b**) 3.89, (**c**) 6.41, (**d**) 8.50. Reprinted with permission from the ref. [58].

The above discussion on the orientation of spheroidal particles applies only to the upper part of the particle assemblies. However, SEM micrographs of fractures in particle assemblies show that the nematic orientation is also maintained deep within the assemblies (see Figure 17).

It is worth noting that nanomechanical and micromechanical studies (using a Berkovich nanoindenter with a tip radius of r ≤ 20 nm and a flat indenter with a diameter of 100 µm) provided the first information on the elastic modulus of a quasi-nematic polymeric colloidal crystalline material. It should be noted that the dimensions of the tips and spheroids indicated that measurements using the Berkovich tip give the elastic modulus of individual spheroids, while the flat indenter with a tip area of approximately 7.9 × 10^3^ μm^2^ affects several thousand spheroids. It was noted that the elastic modulus of individual spheroids was an order of magnitude greater than the elastic modulus of the quasi-nematic colloidal assembly of spheroids.

## 5. Toward the Application of Polymer Spheroidal Nano- and Microparticles

The vast majority of research on polymer nanoparticles and microparticles has focused on their application in medicine, mainly in drug delivery systems. It is worth noting that, according to Web of Science, 925 articles on this topic have been published in the last twelve months. The number of publications in other fields was significantly lower. An example, in the fields of sensors 317, microelectronics 16, and medical diagnostics 12. It is worth mentioning the significant field of mass production of coating materials (847 publications in the last twelve months). However, this topic is beyond the scope of our review.

Bibliometrics reveal the risks associated with bringing a new drug to market. This is suggested by the large number of articles on drug delivery published in a given period (e.g., in the last twelve months) and the extremely small number of publications on phase I clinical trials (925 and 2, respectively). This is predominantly due to the high costs of cell culture research, the enormous costs of animal testing, and the giant costs of clinical trials.

Spheroid polymer particles have only recently attracted the attention of scientists, and none of them have yet been used outside the laboratory. However, several systems are currently under development, as shown below.

The role of the shape anisotropy of the drug carriers has already been recognized in an early review published in 2007 by S. Mitragotri [73]. However, at that time, the review could provide just an introduction to the realm of anisotropic particles rather than a deeper insight.

In 2009, Eniola-Adefeso et al. reported on the preparation of the biodegradable poly(lactic-co-glycolic acid) spheroidal particles loaded with anticancer drug—paclitaxel [29]. Particles were prepared using the oil-in-water (O/W) emulsification-solvent evaporation (ESE) method. Methylene dichloromethane was used as an oil phase. The water phase contained dissolved poly(vinyl alcohol). Formation of spheroidal particles requires proper adjustment of pH, PVA concentration, and the rate of stirring. The authors investigated size, shape, and rate of paclitaxel release. The product was ready for the biological studies.

For various applications, particles are desired that carry specific targeting properties toward the required cancer cells and with enhanced uptake. Recently, Mitragotri et al. revealed that the prolate particles bearing monoclonal antibodies to the cancer cells are by 31% more efficiently uptaken than spherical equivalents with the same volumes [74].

Shape matters not only for nano- and microparticles but also for objects with dimensions in the millimeters range, which could be used as a depot for proteins and cells. Mano et al. have shown it for gel particles fabricated from the photocrosslinkable chitosan [75]. The authors prepared the gel particles by crosslinking droplets of methacrylated chitosan containing BSA as a model protein drug or living cells. Authors noticed close correlation between the particle circularity and the rate of the BSA release, which was slower for particles with higher circularity.

Organic and organic/inorganic conducting and superconducting hybrid compounds are a very interesting class of materials with high potential for application in microelectronics. In 2020 Valade et al. published a review on this subject showing that many of these compounds can be shaped into nanospheroids [76]. Spheroidal particles can be easily deposited from suspension using conventional sputtering or ink-jet printing forming above the percolating threshold, the conducting arrangements. These methods can be used for production of flexible circuits and organic field effect transistors [76].

In some fields of medicine, progress is hampered by the remaining unsolved. Particularly, it is the case of neurodegenerative brain illnesses, when the effective transfer of the bioactive substances through the blood–brain barrier (BB-barrier) remains a challenge. Recently elaborated colloidal unimolecular polymers (CUP) with a few nanometers in size and spheroidal shape would be a good candidate for testing carriers through the BB-barrier [77].

Another important problem that requires special attention is drug resistance to cellular uptake. One solution may be based on the controlled uptake of drug-loaded carriers and fast release on demand. However, the fundamental studies should not be forgotten, because they provide tools and ideas for the applied research. The example we mention is orientation-dependent uptake of spheroidal particles [78].

## 6. Conclusions

Polymer prolate spheroids with colloidal dimensions (from a few nanometers to about 100 μm) are a class of the most investigated anisotropic particles. More information about prolate spheroids is given in the Introduction. According to geometry, the prolate spheroid is formed by the rotation of an ellise along its long axis. However, it should be stressed that in the vast majority of publications, authors use the unequivocal term “ellipsoid” even when from the experimental part it is evident that they produced spheroids. The discrimination between spheroid” and “ellipsoid is important because even when every “spheroid is an “ellipsoid”, not every ellipsoid” is a “spheroid”. Moreover, readers should be careful, because the authors often call produced particles without sufficient justification.

Many methods can be used to obtain polymer prolate spheroidal particles. Section 2 discussed their advantages and limitations. The list of these methods includes stretching a polymer matrix filled with spherical particles, emulsification of polymer solution and solvent evaporation, controlled dispersion polymerization, electrodynamic jetting, adsorption of amphiphilic copolymers on spherical particles and copolymer self-organization processes, by microfluidic methods, deformation of spherical particles by irradiation, and by phase separation processes. The most universal method is the production of the prolate spheroidal particles by stretching the polymer matrix filled with spherical particles and isolation of spheroidal particles. The method provides very good control of particle shape, but its disadvantage consists of a small scale (1 g) of spheroids’ production. However, the process should be easy to scale up. On the other hand, the electrodynamic jetting microfluidic methods are continuous processes, but produce not spheroids but spheroid-like particles.

The significant progress was achieved in the understanding of the behavior of individual spheroidal prolate spheroids on liquid-air interphases (presented in Section 3). Their results were helpful in later self-organization of spheroidal particles into assemblies with controlled architecture. The elaborated methods enabled the preparation of silicon substrates bearing strips regularly distributed the prolate spheroid monolayers containing domains with quasi-nematic colloidal crystalline structures. The width of the strips and the interstrip distance were controlled by particle concentration and ethanol/water composition of the continuous phase of the suspending medium. It has also been shown that evaporation of the liquid phase from concentrated spheroid suspensions produces colloidal assemblies with quasi-nematic lateral arrangement not only at the surface but also in the particle arrays interior. The prolate spheroidal arrays as so-called “superstructures” may be suitable for controlled guiding light and useful as optoelectronic devices with improved photonic and mechanical properties compared to spherical patterns [79].

To date, results of experimental studies on polymer prolate spheroids are in its infancy and are the entrance to a new branch of science. We believe that within a relatively short period the new materials will be developed. They will use the fundamental knowledge on obtaining and behavior of the nano- and microparticles with shape anisotropy, as developed microfluidics and electrohydrodynamic jetting allow their fast, and efficient production. In particular, in biosciences, the ability of the prolate spheroids to enter certain cells more efficiently than spherical particles can be applied in modern drug delivery systems, non-Brownian motion during migration through blood vessels in implanted biosensors, respectively.

In addition, the spheroids able to adjust the shape and size to blood vessels will allow them to be used in various forms of drug delivery systems and implanted biosensors. The delivery systems will release the cargo “on wish” in a target tissue under the influence of a stimulus, with the controlled doses for a designed period of time and degrade gradually till the biologically inert low-molecular-weight products.

The nanostructured polymer spheroidal particles, obtained by diblock copolymer assemblies that can transform shape, may find applications as sensing materials, optical devices, drug delivery systems, and as integral parts of smart materials.

The results of the reviewed papers constitute a basis for research on the preparation of improved devices and tools, and the knowledge gained helps to understand interactions with living organisms.

## Figures and Tables

**Table 1:** Overview of methods leading to obtain polymer prolate spheroidal particles.

Method of Obtaining Particles	Process Parameters	Product Parameters	Remarks	Ref.
Uniaxial stretching of a polymer matrix with particles	Poly(vinyl acetate)	Spheroids AR = 5, AR = 10	Possibility of obtaining spheroids with a controlled and broad range of aspect ratios and sizes from nano- to several microns. “Perfect spheroids” can be obtained. Relatively low yield of the process. Controlled the high temperature of the film elongation. Necessity of washing procedures to remove the polymer matrix.	[16]
	Polystyrene spherical particles, matrix 4–5% PVA/H_2_O	AR = 1.93–5.65		[21]
	Polystyrene spherical particles, liquefaction or stretch of matrix with particles, various T_g_, and thickness of the film	Over 20 various shapes Spheroids—when the ratio of film thickness to microsphere diameter ≥ 40		[22]
	Polystyrene microspheres with hydrophilic shell and polystyrene core, matrix: PVA (10–12%) in H_2_O;	Spheroids, AR = 2.03–6.74		[27]
	Polylactide, Polylactide-co-polyglycolide, matrix: Poly(ethylene-alt-maleic acid) in H_2_O;	Spheroids, AR = 1.93–5.65 Spheroids with COOH groups, AR = 1.1–5.0		[29,30]
	Poly(4-vinyltoluene), Poly(methyl methacrylate), matrix: crosslinked PDMS;	Spheroids, AR = 1.1–7.0;		[18]
	Poly(methyl methacrylate) microspheres, matrix: crosslinked PDMS	Spheroids, AR = 1.1–10.0;		[20]
	Hollow polystyrene microspheres	Spheroids, AR = 1.7–7.7;		[23]
	Polystyrene microspheres with *D*_n_ = 0.5–10.0 μm, strip elongated with gradient strength	Spheroids with variable AR		[24]
Emulsification-solvent evaporation method	Polylactic-co-polyglycolic acid (PLGA)	pH = 8.4, 1% PVA, 1.2% Tris emulsion with PLGA in CH_2_Cl_2_;	High amount of nano- or micrometer-sized spheroids in a short time. Lack of control of the aspect ratio and homogeneity.	[29]
Polymerization of spheroids in dispersion	Glycidyl methacrylate, AIBN, DDMAT, PVP	Spheroids with core–shell morphology, AR = 1.0–3.18;	Spheroids with a limited AR range are obtained; no data on other monomers.	[31]
Electrohydrodynamic jetting	Polystyrene with acetone/H_2_O, toluene	Spheroids swollen with toluene, AR = 1.5–2.4;	Continuous process, conversion spheres to spheroids in one step; a large quantity of particles in a short time; limited range of AR.	[32,33]
Adsorption of amphiphilic copolymers on solid particles	Polystyrene_156_-b-polyacrylic acid_10_ adsorber on silica spheres (70–130 nm)	Spheroid-like core–shell particles with a size depending on the size of the silica particles	Non-uniform particles are obtained; two or more silica particles in one prolate spheroidal particle.	[34]
Self-organized copolymers	Polystyrene_9.8k_-b-poly-4-vinylpyridine_10k_ copolymer—reversible change in morphology depending on the presence of PVA	Spheroids with the lamellar domains, micrometer-sized	Spheroids’ shape is reversed. Transformation of shape assisted by solvent annealing. The AR is barely controlled.	[43]
	(P(NB-g-PLLA)-*b*-P(NB-g-PS) bottle brush copolymer	Spheroids with lamellar domains with AR = 1.5–2.8.		[44]
Microfluidic methods	Monomers’ droplets solidification in situ. A broad spectrum of used monomers	Spheroids with various sizes and internal morphology. Size depends on the channel geometry.	An efficient method to produce spheroids with very narrow dispersity and shape control. Possibility of a broad range of AR. Spheroids size 20–1000 μm.	[17,35]
Obtaining spheroids by irradiation	Spherical particles (160–310 nm) carrying photosensitive ligands	Spheroids formed by UV light.AR density of chromophores dependent.	Limited control over the aspect ratio of spheroids. Shape transformation is reversible.	[36]

## Data Availability

No new data were created or analyzed in this study. Data sharing is not applicable to this article.
